# Improving tumor microenvironment assessment in chip systems through next-generation technology integration

**DOI:** 10.3389/fbioe.2024.1462293

**Published:** 2024-09-25

**Authors:** Daniela Gaebler, Stephanie J. Hachey, Christopher C. W. Hughes

**Affiliations:** ^1^ Molecular Biology and Biochemistry, University of California, Irvine, Irvine, CA, United States; ^2^ Biomedical Engineering, University of California, Irvine, Irvine, CA, United States

**Keywords:** cancer immunology, tumor microenvironment, next-generation technology, tumor-on-chip, bioengineering, biosensors, sequencing, bioprinting

## Abstract

The tumor microenvironment (TME) comprises a diverse array of cells, both cancerous and non-cancerous, including stromal cells and immune cells. Complex interactions among these cells play a central role in driving cancer progression, impacting critical aspects such as tumor initiation, growth, invasion, response to therapy, and the development of drug resistance. While targeting the TME has emerged as a promising therapeutic strategy, there is a critical need for innovative approaches that accurately replicate its complex cellular and non-cellular interactions; the goal being to develop targeted, personalized therapies that can effectively elicit anti-cancer responses in patients. Microfluidic systems present notable advantages over conventional *in vitro* 2D co-culture models and *in vivo* animal models, as they more accurately mimic crucial features of the TME and enable precise, controlled examination of the dynamic interactions among multiple human cell types at any time point. Combining these models with next-generation technologies, such as bioprinting, single cell sequencing and real-time biosensing, is a crucial next step in the advancement of microfluidic models. This review aims to emphasize the importance of this integrated approach to further our understanding of the TME by showcasing current microfluidic model systems that integrate next-generation technologies to dissect cellular intra-tumoral interactions across different tumor types. Carefully unraveling the complexity of the TME by leveraging next generation technologies will be pivotal for developing targeted therapies that can effectively enhance robust anti-tumoral responses in patients and address the limitations of current treatment modalities.

## 1 Introduction

Cancer is the second leading cause of death worldwide and cases are expected to exceed 2 million in the US alone in 2024 (Sung et al., 2021; Siegel et al., 2024), with the global cancer burden expected to further increase to 28.4 million cases in 2040 (Sung et al., 2021). As a result, extensive efforts have been undertaken to deepen our understanding of the genetic and molecular characteristics of this multifaceted disease. Cancer is described as a dynamic evolutionary process characterized by continuous interactions between tumor cells and the surrounding tumor microenvironment (TME) (Anderson and Simon, 2020; De Visser and Joyce, 2023). The TME is a complex, constantly evolving entity and its composition varies between tumor types (Anderson and Simon, 2020). Besides cancerous cells, hallmark features of tumors encompass various components such as the extracellular matrix (ECM), endothelial cell (EC)-lined blood vessels and lymphatics, and other non-cancerous cell types such as cancer-associated fibroblasts (CAFs), mesenchymal stromal cells, pericytes, dendritic cells (DCs), macrophages, myeloid-derived suppressor cells (MDSCs), mast cells, polymorphonuclear cells, natural killer cells (NKs), and T and B lymphocytes (Baghban et al., 2020; Anderson and Simon, 2020; De Visser and Joyce, 2023). Complex tumor-stromal, tumor-immune and immune-stromal interactions in the TME lead to the secretion of a myriad of growth factors, cytokines, chemokines, enzymes, and extracellular vesicles. These components play a pivotal role in modulating tumor status by regulating the activation of diverse metabolic signaling pathways and inflammatory responses (Baghban et al., 2020; Hachey et al., 2021; Fontana et al., 2021; De Visser and Joyce, 2023; Hachey et al., 2024; Gaebler et al., 2024). Accordingly, cellular crosstalk within the TME contributes to tumor progression, metastasis and therapy resistance, often by impacting vascularization (McMillin et al., 2013; Fontana et al., 2021; Rodrigues et al., 2021; Xiao and Yu, 2021; Hachey et al., 2024; Gaebler et al., 2024). Specifically, CAFs produce large amounts of collagen cross-linking enzymes and ECM-degrading proteases that modify the mechanical characteristics of tumors and influence the process of angiogenesis (Walker et al., 2018; Nissen et al., 2019; Belhabib et al., 2021; Hachey et al., 2024).

Angiogenesis is required for supplying the tumor with sufficient nutrients, metabolites and oxygen. However, the imbalance of pro-angiogenic and anti-angiogenic signaling molecules present in the TME often leads to the formation of abnormal, leaky blood vessels that promote increased interstitial fluid pressure and, consequently, nutrient and oxygen deprivation in some tumor areas (Baghban et al., 2020; Yang et al., 2024). Tumor hypoxia, recognized as an important contributor to the poor therapeutic efficacy of current anti-angiogenic drugs (Jiang et al., 2020; Kierans and Taylor, 2021), actively promotes tumor angiogenesis. This process accelerates tumor growth and metastasis by stimulating the expression of epithelial-mesenchymal transition (EMT) markers (e.g., N-cadherin) (Cheng et al., 2011; Azab et al., 2012; Jiang et al., 2020). Additionally, it induces the production of matrix-altering metalloproteinases that further promote invasive metastasis (Zhao et al., 2014; Revuelta-López et al., 2013), along with inducing metabolic changes leading to increased glucose uptake and glycolysis (Leung et al., 2017; Kierans and Taylor, 2021). Increased glycolysis in the TME in turn leads to increased tumor acidification, which further creates an immunosuppressive environment by suppressing immune cell activity and attracting immunosuppressive immune cell populations into the TME (Kierans and Taylor, 2021; Ren et al., 2022). These immunosuppressive cells, including MDSCs, Tregs and tumor-associated macrophages (TAMs), shape the TME by secreting immunosuppressive cytokines and chemokines, thereby contributing to tumor immune evasion and limiting therapy effectiveness (Kierans and Taylor, 2021; Ren et al., 2022; Fang et al., 2023).

In essence, the TME coordinates a multifaceted interplay involving cellular dynamics, vascular development, tissue rigidity, hypoxia, and acidification, all of which influence tumor behavior. Accurately modeling these key aspects of the TME in relevant physiological models is essential not just for enhancing our comprehension of the intricate cellular interactions within the TME, but also for translating fundamental biomedical research into the development of therapeutics customized to individual patients. Traditionally, approaches to elucidate the cellular, biochemical and biophysical interactions in the TME have heavily relied on both *in vivo* animal models and static *in vitro* models, such as 2D cell monolayers (Fontana et al., 2021; Barozzi and Scielzo, 2023). However, the limited applicability of conventional cell cultures or animal models to human diseases has constrained their effectiveness in the development of new cancer treatments for patients (Mak et al., 2014; Sun et al., 2022; Barozzi and Scielzo, 2023). This is evident in the observation that 90% of drugs progressing through phase I clinical trials fail, primarily due to lack of efficacy, followed by unmanageable toxicity (Sun et al., 2022).

In contrast to traditional model systems, microphysiological systems (MPS) offer a means to reconcile the balance between experimental precision and physiological relevance. MPS, or “organ-on-a-chip” devices, have emerged as a breakthrough technology by combining microfluidic technology and three-dimensional (3D) cell culture techniques to replicate the complexity and attributes of human organs on a microscale (Leung et al., 2017; Fontana et al., 2021; Rodrigues et al., 2021). Through precise microfluidic engineering, these devices can replicate the dynamic physiological features found in tissues, including physiological flow and shear stress, transport of nutrients, metabolites and gasses, and cell-cell interactions, within an environment with high spatiotemporal control (Jensen and Teng, 2020; Fontana et al., 2021; Rodrigues et al., 2021; Hachey et al., 2021; Hachey et al., 2023a; Hachey et al., 2024). To date, MPS models have evolved to mimic various human tissues and organs, leveraging their adaptable structural design to enable the development of “multi-organ-on-a-chip” and “body-on-chip” systems (Edington et al., 2018; Kimura et al., 2018; Picollet-D’hahan et al., 2021). MPS models are invaluable in studying numerous malignancies, including cancer, by accurately replicating tissue architecture (Huh, 2015; Seiler et al., 2020; Rodrigues et al., 2021; Hachey et al., 2021; Baptista et al., 2022). They enable cellular crosstalk across multiple cell types and faithfully reproduce a spectrum of biological, physical, morphological, structural, mechanical, and biochemical cues including tissue stiffness, desmoplasia, angiogenesis, hypoxia and tumor acidification (Huh, 2015; Hachey et al., 2021; Lam et al., 2021; Rodrigues et al., 2021; Baptista et al., 2022; Gaebler et al., 2024; Hachey et al., 2024; Bayona et al., 2024). Moreover, MPS offer promising avenues for drug discovery and testing by faithfully mirroring human drug responses under various physiological conditions (Benam et al., 2016; Rodrigues et al., 2021; Hachey et al., 2021; Moon et al., 2023; Hachey et al., 2024). Despite the potential of microfluidic chips to be valuable preclinical tools for advancing our understanding of tumor pathology and discovering new strategies to improve the TME, few research groups have incorporated next-generation technologies into their designs.

Numerous concepts have emerged for the evolution of MPS. These concepts encompass a range of innovations, spanning from automation improvements and the integration of intelligent readout systems to the adoption of bioprinting methods, advanced imaging techniques, single-cell and next-generation sequencing approaches, and biosensor integration. The overarching aim of these approaches is to advance drug development, refine toxicity assessment, and improve the accuracy of disease modeling by enhancing fluid dynamics, increasing design flexibility, and optimizing overall system functionality (Dababneh and Ozbolat, 2014; Fang et al., 2022; Liu et al., 2022; Deliorman et al., 2023). For example, contemporary bioprinting techniques enable precise spatial cellular organization with enhanced complexity (Dababneh and Ozbolat, 2014; Liu et al., 2021; Deliorman et al., 2023), while biosensors can monitor levels of oxygen, H+, or glucose (Bhalla et al., 2016; Ferrari et al., 2020). Additionally, sequencing technology assists in analyzing disease-related signaling pathways and mechanisms of drug resistance (Hachey et al., 2024). Integrating these next-generation technologies with microfluidic platforms holds immense potential for advancing biomedical research by providing comprehensive insights into the intricate interplay of biological, physical and biochemical cues within the TME. In this review, we delve into existing MPS designed to analyze various aspects of the TME and discuss ongoing efforts to integrate these models with next-generation technologies.

## 2 Defining the tumor microenvironment

**FIGURE 1:**
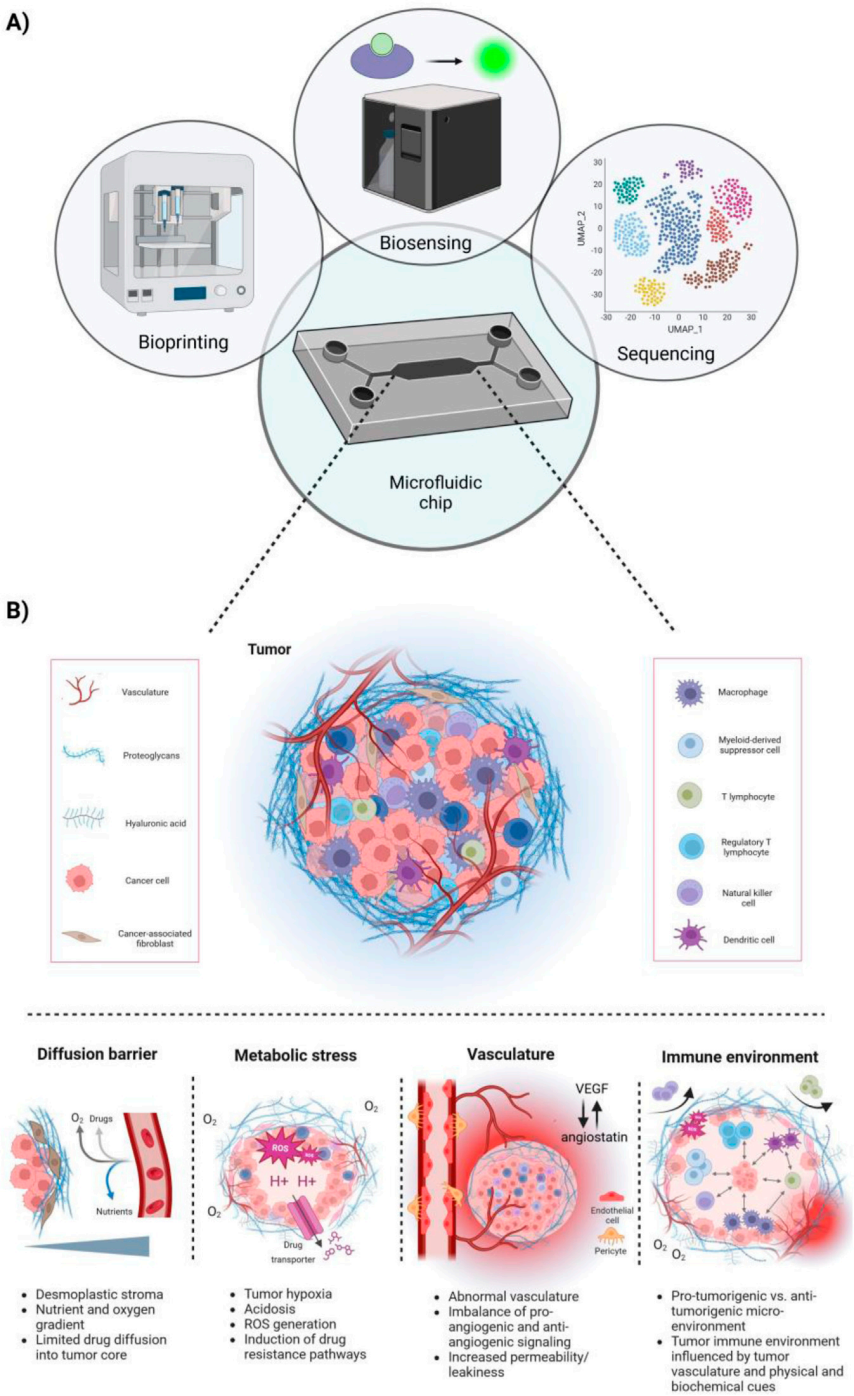
Microfluidic chips integrating next-generation technologies allowing for a more holistic approach to model the complex tumor microenvironment (TME). **(A)** Schematic overview of microfluidic chips integrating next-generation technologies, such as bioprinting, biosensing and cell sequencing. **(B)** Overview of key features of the TME. Tumors are characterized by a desmoplastic stroma, high metabolic stress, including tumor hypoxia and acidosis, and abnormal tumor vasculature. This correlates with the tumors’ limited perfusion with nutrients and oxygen and further promotes the induction of drug resistance pathways. Further, the tumor-immune microenvironment is directly influenced by the tumor vasculature and biophysical and biochemical cues, creating either a pro-tumorigenic or anti-tumorigenic milieu. Typically, an immuno-suppressed environment lacks the presence or activity of NK cells, T effector cells and M1 macrophages, whereas immunogenic tumors exhibit the opposite phenotype.

Solid tumors consist of an abnormal mass of cells including blood vessels, lymphatic vessels, ECM components, cancer stem cells (CSCs) and a variety of stromal and immune cells. Once they surpass a few cubic millimeters, they require angiogenesis—a process involving the creation of new blood vessels from existing vascular beds—to acquire the nutrients essential for their heightened energy demands and growth. In non-pathological angiogenesis, mature vessels form tight endothelial junctions, with pericyte and smooth muscle cell coverage, ensuring vascular stability and blood perfusion (Adams and Alitalo, 2007; Chung et al., 2010; Carmeliet and Jain, 2011). In contrast, pathological tumor angiogenesis results from an imbalance between pro-angiogenic and anti-angiogenic signaling in the TME. Pro-angiogenic factors like VEGF-A, bFGF, and IL-8 become abundantly present in the TME, overwhelming angiostatic signals such as angiostatin, endostatin and TSP-1; leading to a pro-angiogenic switch. This imbalance causes the network of tumor-associated blood vessels to be structurally and functionally abnormal, characterized by disrupted, immature, chaotic, and ill-perfused vessels, hindering nutrient delivery and fostering tumor growth and metastasis (Chung et al., 2010; Carmeliet and Jain, 2011; De Palma et al., 2017). This results in several downstream effects (Figure 1).

Insufficient pericyte coverage and loose inter-endothelial cell junctions result in leaky vessels, and in combination with dysfunctional lymphatic vessels increase intra-tumoral interstitial fluid pressure. Interstitial hypertension can not only promote tumor growth and metastasis but can also present a substantial barrier for drug delivery and therapy response, particularly in solid tumors (Stylianopoulos, 2017; Stylianopoulos et al., 2018). Further, compressed blood vessels lead to hypoperfusion and hypoxia, which promote tumor progression and therapy resistance via induction of TGF-β-dependent and Snail-dependent EMT (Lundgren et al., 2009; Stylianopoulos, 2017; Tam et al., 2020; Khouzam et al., 2022). In addition, both tumor hypo-perfusion and interstitial hypertension can lead to a desmoplastic reaction in some tumors, such as pancreatic, prostate, cervical or colorectal cancers (Neesse et al., 2015; Da Silva et al., 2015; Rice et al., 2017; Ueno et al., 2021; Wolf et al., 2023). Desmoplasia is characterized by an excessive accumulation of tumor ECM, changes in stromal cell proliferation and the activation of fibroblasts (Nissen et al., 2019; Xiao et al., 2023). Cancer cell-mediated signaling, including via TGF-β and other growth factors, to fibroblasts can reprogram them into CAFs (Shi et al., 2020; Watabe et al., 2023). The accumulation of CAFs in the TME is associated with the increased synthesis and cross-linking of collagen, ultimately contributing to a positive feedforward loop that drives fibrosis, hypoxia, tumor progression, tumor metastasis, immune cell exclusion and immunotherapy resistance (Nissen et al., 2019; Shin et al., 2019; Shi et al., 2020).

In the TME, continuous, uncontrolled, and disorganized cell proliferation can quickly surpass the supply of oxygen and nutrients. The poorly formed and collapsed blood vessels cannot meet these high demands, leading to hypoxic regions, increased ECM deposition, heightened cancer cell glycolysis, and acidification of the TME (Khouzam et al., 2022). Hypoxia is positively associated with the hallmarks of tumorigenesis, as it promotes the progression of solid tumors by driving tumor heterogeneity, plasticity, stemness, genetic instability and aggressiveness by modulating autophagic and metabolic processes (Rakotomalala et al., 2021; Khouzam et al., 2022). Underlying mechanisms by which hypoxia influences metabolic pathways in the TME are mainly mediated by members of the hypoxia inducible factor (HIF) family of transcription factors (Samanta and Semenza, 2018; Khouzam et al., 2022). This family controls the expression of genes involved in glycolysis, pH regulation, wound healing, angiogenesis and other pro-tumorigenic processes, such as ECM remodeling (Kierans and Taylor, 2021; Lee et al., 2021; Khouzam et al., 2022).

Metabolic adaptations are necessary to sustain cell viability and proliferative potential despite low oxygen and nutrient availability in the hypoxic TME. In particular, low cellular oxygen levels lead to a switch from oxidative phosphorylation to glycolysis in order to generate sufficient adenosine triphosphate (ATP) (Samanta and Semenza, 2018; Kierans and Taylor, 2021; Khouzam et al., 2022). This switch is mediated by HIF-1α, which induces the upregulation of glucose transporters and key regulatory glycolytic enzymes, while simultaneously inhibiting components of the tricarboxylic acid (TCA) cycle (Samanta and Semenza, 2018; Khouzam et al., 2022). While these glycolytic processes lead to the acidification of the cell due to the accumulation of the byproducts lactate and H+, HIF-1α induces the expression of carbonic anhydrases and transporters to remove these byproducts (Chiche et al., 2010; Samanta and Semenza, 2018; Khouzam et al., 2022). Consequently, the TME is depleted of glucose, enriched in lactate and characterized by an acidic pH. These features promote the function of immunosuppressive cells, while suppressing anti-tumorigenic processes (Samanta and Semenza, 2018; Khouzam et al., 2022); ultimately shaping the tumor immune microenvironment into an immunologically cold milieu.

The ECM, divided into the interstitial matrix and the basement membrane, is a major component of the TME and serves a reservoir of bioactive molecules that can induce intra-cellular signaling to regulate growth, survival, differentiation, migration and immunity (Pickup et al., 2014; Nissen et al., 2019; He et al., 2021). It constitutes a 3D, non-cellular network composed of elastin, fibronectin, laminins, collagens, proteoglycans, glycosaminoglycans and other glycoproteins, whereby collagens are the most abundant component (Pickup et al., 2014; Nissen et al., 2019; He et al., 2021). Importantly, dysregulation of tumor-induced ECM remodeling or degradation can drive tumor progression by fostering tumor growth, invasion, metastasis, and angiogenesis (Pickup et al., 2014; Nissen et al., 2019). In particular, tumor-associated ECM is characterized by the abnormal production and quantity of ECM constituents, such as structurally and biochemically aberrant collagens, altered mechanical properties and functional alterations, such as transformed mechanosignaling routes (Pickup et al., 2014; Nissen et al., 2019). Invading cancer cells release ECM remodeling enzymes, including matrix metalloproteinases (MMPs) and plasminogen activators. For instance, patients with invasive breast cancer were found to have significantly elevated serum levels of MMP-2 (Sheen-Chen et al., 2001). Thicker, denser and more organized collagen fibers have been reported in some tumors (e.g., pancreatic, gastric or breast tumors) (Zhou et al., 2017; Rice et al., 2017; Koorman et al., 2022). This dense fibrotic tumor ECM promotes cancer invasion and plays a significant role in physically excluding anti-tumor immune cells (Cao et al., 2016; Mariathasan et al., 2018; Belhabib et al., 2021; Flies et al., 2023). It also actively interacts with immune cells in the TME, regulating their activity through adhesive binding interactions, such as the dysregulation of cell adhesion receptors like integrins, and direct interactions with immunostimulatory or immunoinhibitory receptors on the immune target cells (Peng et al., 2020; Larsen et al., 2020; Flies et al., 2023; Zhang et al., 2023).

Hypoxia and acidosis, resulting from abnormal tumor vasculature, attract immunosuppressive cells, diminish effector T cell activity, and further impede therapeutic delivery and efficacy (Hu et al., 2021; Khouzam et al., 2022; Mortezaee et al., 2023). Specifically, hypoxic tumor cells outcompete various immune cells, including cytotoxic T lymphocytes (CTLs), macrophages, NK cells and DCs, for glucose, which negatively affects their activation, anti-tumorigenic function and differentiation (Youssef et al., 2023). High lactate levels and low pH in the TME lead to decreased NK cell cytokine production and suppressed cytotoxicity (Husain et al., 2013; Terrén et al., 2019), while CTLs show reduced survival, function and migratory behavior (Haas et al., 2015; Quinn et al., 2020; Elia et al., 2022; Youssef et al., 2023). Lactate further disturbs DC maturation (Gottfried et al., 2006), increases the levels of MDSCs (Husain et al., 2013), enhances the function of immunoregulatory Tregs (Gu et al., 2022) and induces the polarization of tumor-associated macrophages (TAMs) into the immunosuppressive M2-like phenotype in the TME (Zhang et al., 2021; Tao et al., 2023). In addition, hypoxia also plays a crucial role in disrupting lipid and amino acid metabolism, stimulating the expression of TGF-β and VEGF and inactivating the acid-labile, T cell derived cytokine IFN-γ; thereby further shaping the immunosuppressive status of the tumor (Khouzam et al., 2022). VEGF, TGF-β, and IFN-γ are key players in orchestrating critical molecular processes in the tumor that eventually influence immunotherapy response. While VEGF mainly regulates angiogenesis and thereby contributes to tumor hypoxia-related mechanisms (Khouzam et al., 2022), TGF-β regulates cancer cell proliferation, suppresses immune cell functions, promotes the conversion of fibroblasts into myofibroblasts, contributes to EMT, drives the overproduction of ECM and promotes angiogenesis (Ibi et al., 2024; Watabe et al., 2023; Deng et al., 2024). IFN-γ is mainly secreted into the TME by NK cells and cytotoxic T cells, whereby it can lead both to the upregulation of MHC class I molecules, leading to an increased antigen presentation that is advantageous for T cell activation (Zhang et al., 2019; Qian et al., 2018; Khouzam et al., 2022). Besides upregulating PD-L1 on cancer cells, hypoxia also regulates the expression of PD1, CTLA4, CD47, T-cell immunoglobulin and mucin domain 3 (TIM3) and lymphocyte activation gene 3 (LAG3), all of which can interfere with the anti-tumor function of effector T cells (Hu et al., 2021; Mortezaee et al., 2023). Notably, the upregulation of immune checkpoints in hypoxic tumors is typically associated with a poor prognosis (Hu et al., 2021; Mortezaee et al., 2023). Immune checkpoint inhibitor (ICI) therapy approaches, such as anti-PD-1, anti-PD-L1 or anti-CTLA-4 antibodies, have been designed to reduce T cell dysfunction/exhaustion and have led to significant improvements in some patient populations (Barbari et al., 2020; Ren et al., 2022; Ma et al., 2023). Further, limiting hypoxia in the TME improves the response to ICIs, suggesting that strategies to modulate oxygen levels could enhance the efficacy of cancer immunotherapies in the future (Luo et al., 2022).

An aberrant tumor vasculature can act as a functional barrier, regulating the infiltration and activity of immune cells, including T cells, into the TME. Importantly, recent studies have linked the level of tumor vascularization to the response to immunotherapy and other therapeutic treatments (Schaaf et al., 2018; Ollauri-Ibanez et al., 2021). In the realm of immunotherapy, an adequate vascular network within the tumor can facilitate the influx of immune cells, including T cells, to the TME. This, in turn, can enhance the effectiveness of immunotherapeutic interventions like ICIs by improving T cell infiltration into the tumor and bolstering their anti-tumor function. Concurrently, aberrant vasculature establishes a physical barrier that impedes T cell infiltration (Schaaf et al., 2018; Bruni et al., 2023). ECs within tumor blood vessels actively suppress anti-tumor immunity by inhibiting the recruitment, adhesion, and activity of immune cells via hypoxia-induced upregulation of VEGF-A, IL-10, and prostaglandin E2 (PGE2); collectively inducing FasL expression on tumor ECs and triggering the apoptosis of T cells upon binding to Fas (Motz et al., 2014; Zhang et al., 2022). Additionally, tumor-associated ECs can selectively enhance the recruitment of Tregs through the upregulation of the multifunctional endothelial receptor CLEVER-1/stabilin-1, suggesting that tumor endothelium supports both the recruitment and survival of immunosuppressive T cells (Fang et al., 2023). Furthermore, VEGF-A induces a clustering defect in adhesion molecules like intercellular adhesion molecule (ICAM)-1 and vascular cell adhesion protein (VCAM)-1, impeding immune cell extravasation. Therefore, in addition to its role in stimulating angiogenesis, VEGF-A contributes to the hindrance of efficient EC–lymphocyte interaction, preventing CD8^+^ CTLs from reaching the tumor and distributing within the TME (Schaaf et al., 2018; Jain, 2003).

Therapeutically restoring the structural and functional integrity of tumor blood vessels provides a promising strategy to enhance both drug and immune cell delivery to the tumor, ultimately promoting a beneficial microenvironment through improved blood flow (Carmeliet and Jain, 2011; Mpekris et al., 2020). Traditional anti-angiogenic approaches that target tumor-associated vasculature to starve tumor cells have often failed as single agent treatments, leading to increased tumor hypoxia, drug resistance, and metastasis. Consequently, various strategies have emerged to normalize tumor-associated vasculature, allowing better perfusion of nutrients and immune cells, especially in combination with immunotherapy. New insights into vascular and immune normalization strategies hold promise for improving cancer therapy outcomes, including methods targeting VEGF signaling, Ang-Tie signaling, oncogenic signaling in cancer cells, and even CD4^+^ T-cells (Schmittnaegel et al., 2017; Choi and Jung, 2023). Combining these approaches may yield even greater efficacy in cancer treatment, particularly for tumors with a low immune response.

## 3 Tumor chips model key characteristics of the TME

Three-dimensional microfluidic-based human tumor models, also called “cancer-on-chip” or “tumor chips”, have been developed to investigate the TME with the ultimate goal to create reliable human model systems with high clinical relevance. Unlike traditional methods, microfluidic technology provides several advantages, such as precise control over chemical and physical parameters at the micrometer scale, versatility in oncology research, and the capability to observe biological processes with high spatiotemporal resolution (Piccolo et al., 2021; Liu et al., 2022; Zhou et al., 2023; Jouybar et al., 2024). Tumor chips comprise cancer cells, cancer spheroids or organoids, often with associated stroma, in a 3D hydrogel matrix under dynamic flow conditions (Piccolo et al., 2021; Gaebler et al., 2024). These models have been developed to replicate a wide range of primary cancer types, spanning from carcinoma of the lung, breast, stomach, colon and rectum, prostate, esophagus, pancreas, liver, ovary, skin, and brain (among other cancers), as well as cancer metastasis to bone, liver, brain, peritoneum and lung (Hsiao et al., 2009; Bersini et al., 2014; Chen et al., 2016; Kocal et al., 2016; Khazali et al., 2017; Aleman and Skardal, 2019; Oliver et al., 2020; Hachey et al., 2021; Straehla et al., 2022; Ibrahim et al., 2022; Das et al., 2022; Haque et al., 2022; Flont et al., 2023; Shen et al., 2023; Hachey et al., 2024). Importantly, these models not only allow for the accurate reproduction of spatial aspects of the *in vivo* tumor architecture (Hachey and Hughes, 2018), but also faithfully replicate cancer cell gene expression patterns (Edmondson et al., 2014; Hachey et al., 2023a). Multiple studies have assessed critical aspects of the TME that foster or inhibit tumor growth, invasiveness, metastasis, and therapy response (McMillin et al., 2013; Fontana et al., 2021; Rodrigues et al., 2021; Xiao and Yu, 2021). Many of these features are challenging to replicate in conventional 2D cell culture and difficult to individually examine in animal models, emphasizing the importance of tumor chip models for unraveling the impact of individual variables on cancer progression in the highly interconnected TME (Mak et al., 2014; Tsai et al., 2017; Zhou et al., 2023). As a result of their unique potential, tumor chips have undergone wide-ranging refinements and gradual improvements in their complexity, allowing for the incorporation of tumor vasculature, stromal components and immune cells (Piccolo et al., 2021; Jouybar et al., 2024). Additionally, tumor chips incorporate physiological stimuli like mechanical forces, electrical stimulation, and biochemical signals. They are particularly well-suited for integrating next-generation technologies such as 3D bioprinting, single-cell analysis, and biosensing (Tsai et al., 2017; Piccolo et al., 2021; Liu et al., 2022; Zhou et al., 2023).

**FIGURE 2:**
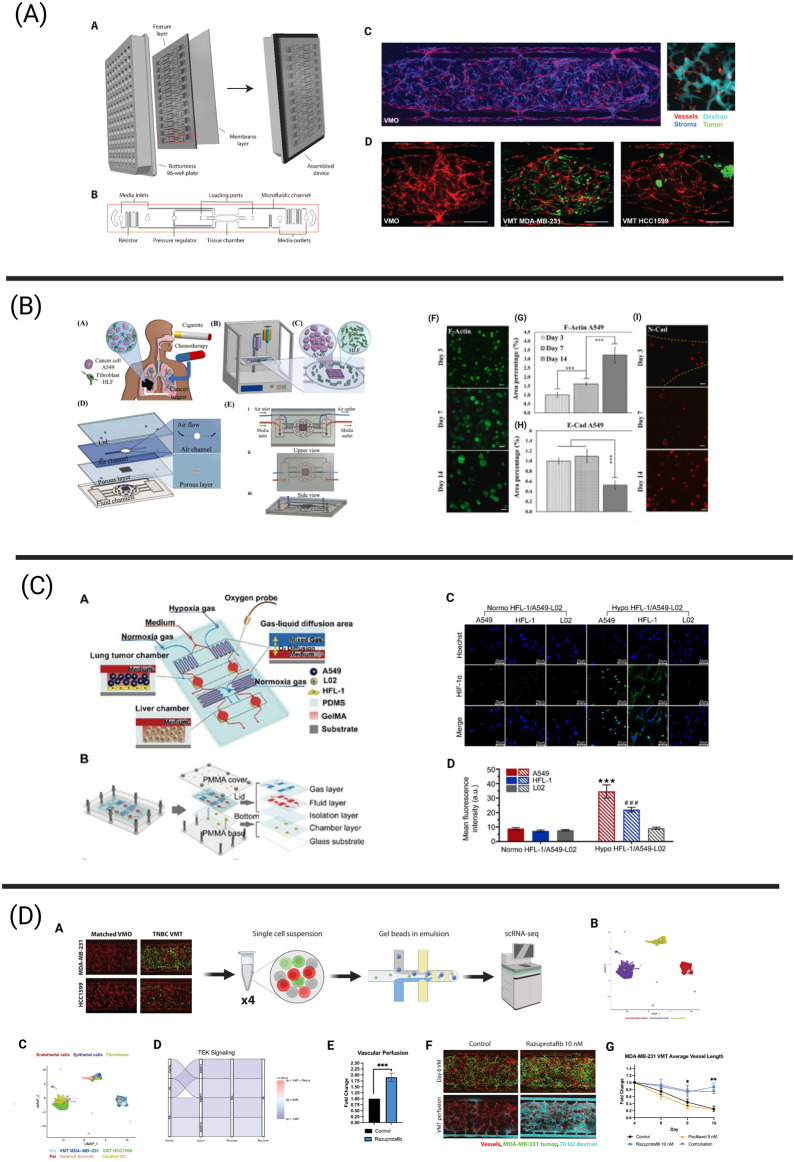
Tumor chips integrating essential components of the tumor microenvironment (TME) via next-generation technologies including bioprinting, biosensing, and single-cell sequencing. **(A)** The vascularized micro-tumor (VMT) represents a physiologically relevant tumor chip model and has been adapted to model a variety of human cancers, including breast cancer. The vasculature is fully perfusable and allows for the delivery of oxygen and nutrients to the tumor. This platform has been adapted to a 96-well plate format. Adapted from Hachey et al. (2023a), Hachey et al. (2024). **(B)** Bioprinting process of microfluidic chip enabling the co-culture of human lung fibroblasts and A549 lung cancer cells to assess the effects of cigarette smoke extract and chemotherapeutics on the development of lung cancer metastatic processes. A549 spheroids gradually increased in size over the culture period of 14 days, resulting in limited oxygen and nutrient availability in the spheroid core. Further, measurement of E-cadherin and N-cadherin expression revealed gradual changes in mesenchymal transition mechanisms. Adapted from Das et al. (2022). **(C)** Schematic overview of a microfluidic chip harboring both a lung and liver compartment and aiming to investigate hypoxia-induced lung cancer metastasis to the liver. An optical biosensor was used to measure oxygen levels in the device. Immunofluorescence revealed increased HIF-1*α* protein expression in A549 and HFL-1 cells cultured under hypoxic conditions in comparison to normoxic conditions. Reprinted (adapted) with permission from Zheng et al. (2021) Copyright (2021) American Chemical Society. **(D)** The schematic illustrates the workflow for scRNA-seq from breast cancer-derived VMTs and matched vascularized micro-organs (VMOs) - tissue constructs without tumors. Cell clusters from VMTs integrate seamlessly with cell clusters from clinical specimens. Downregulated TEK/Tie2 signaling, observed both in the VMTs and in clinical specimens, was restored by treatment with razuprotafib, a VE-PTP inhibitor that activates Tie2. Razuprotafib enhanced vascular perfusion in the VMTs and increased tumor sensitivity to low-dose paclitaxel. Adapted from Hachey et al. (2024).

Tumor chips can replicate numerous aspects of the TME. These include cell-cell and cell-ECM interactions, mechanical forces within the ECM, desmoplasia, gradients of nutrients, pH, and soluble factors, central hypoxia, and abnormal tumor vasculature (Table 1) (Koens et al., 2020; Miller et al., 2020; Jouybar et al., 2024). The vasculature plays a crucial role in the TME and is important for tumor chips to create a physiologically accurate barrier, enable a more reliable assessment of drug delivery and effectiveness, and assess the interaction, function, and extravasation of immune cells. For example, our group has invented a tumor chip that allows for a comparably high level of cellular complexity and includes a functional vasculature (Sobrino et al., 2016; Hachey et al., 2023a). Particularly, this model facilitates the development of “vascularized micro-tumors” (VMTs) by coculture of ECs, fibroblasts and cancer cells under dynamic flow conditions (Figure 2). This environment enables the *de novo* formation of perfusable microvascular networks surrounding the micro-tumors, which mimics the nutrient and therapeutic supply provided by tumor-associated vessels *in vivo* (Sobrino et al., 2016). The VMT model has been adapted to model multiple cancer types with various levels of vascular disruption, such as breast cancer and CRC (Sobrino et al., 2016; Hachey et al., 2021; Jahid et al., 2022; Hachey et al., 2024). It has also been used for drug sensitivity testing on primary, patient-derived CRC cells (Hachey et al., 2023b) and to demonstrate T cell extravasation from vessels into the perivascular space (Hachey et al., 2023a). Moreover, the model has been combined with single-cell RNA sequencing (scRNA-seq) to reveal how tumor-stromal cellular interactions influence the progression and therapy response in triple-negative breast cancer (TNBC) (Hachey et al., 2024). Another MPS model incorporating a functional vasculature has been developed by Shirure and team, who established a perfused 3D microvascular network that supplied nutrients to an adjacent tumor compartment (Shirure et al., 2018). This compartment contained either breast or CRC cell lines, or primary breast tumor organoids that could be cultured in their device for several weeks. Besides assessing tumor growth, sprouting angiogenesis and tumor invasion, the researchers also demonstrated the feasibility of their platform for drug screening studies. In addition to these models, many other tumor chips have been developed, encompassing features such as tumor vasculature (Michna et al., 2018; Yu et al., 2019; Nashimoto et al., 2020), stromal cells (Sobrino et al., 2016; Yu et al., 2019; Ibrahim et al., 2022; Hachey et al., 2023a; Hachey et al., 2024), or immune cells (Kim et al., 2019; Boussommier-Calleja et al., 2019; Kim et al., 2022). These systems enable multicellular crosstalk and/or incorporate physiologically relevant vascularization.

**TABLE 1:** Examples of tumor chips recapitulating key aspects of the tumor microenvironment including hypoxia, acidosis, desmoplasia, vascularization and intratumoral, cellular interactions.

TME feature	Cancer type	Microfluidic device	Cell types	Key findings	Source
Vascularization	Breast cancer, colon cancer	Microfluidic chip with one central tumor spheroid channel, flanked by two EC lined channels	HUVECs, NHLFs, MCF-7 or MDA-MB-231 or SW620 cells	Microfluidic device integrating tumor spheroids and a perfusable vascular network supplying the tumor with nutrients, oxygen and drugs allowing for cell proliferation and survival	Nashimoto et al. (2020)
Vascularization	Breast cancer	Various microfluidic chip designs	telomerase immortalized microvascular endothelial (TIME) cells, MDA-MB-231 cells	Microfluidic platform allowing for complex vascularization to study cell-cell interactions between vasculature and tumor cells	Michna et al. (2018)
Vascularization, tumor-stromal interactions	Ovarian/Omental/peritoneal cancer	Microfluidic device with one cell channel and three fluid channels	ECs, adipocytes, mesothelial cells, SKOV3 or OVCAR3 or OV90 cells	Vascularized model of the peritoneal omentum demonstrating the effect of stromal cells on tumor cell attachment and growth	Ibrahim et al. (2022)
Vascularization, tumor-stromal interactions	Colorectal cancer, breast cancer, melanoma	Microfluidic device with 3 diamond-shaped tissue chambers supplied by two media channels	ECFC-ECs, NHLFs, SW620, SW480, HCT116, MDA-MB-231, MCF-7, MNT-1	Establishment of a tumor chip model incorporating colorectal cancer/breast cancer/melanoma recapitulates tumor metabolic heterogeneity and response to standard-of-care drugs	Sobrino et al. (2016)
Vascularization, tumor-stroma interactions	Colorectal cancer	Microfluidic device with one cell chamber supplied by two media channels	ECFC-ECs, NHLFs, primary patient-derived tumor cells	Tumor chip model incorporating primary colorectal cancer cells mimics histology, tumor growth, metabolic heterogeneity, and drug sensitivity observed in CRC tumors	Hachey et al. (2023a)
Vascularization, tumor-stromal interactions	Breast cancer, colorectal cancer	Microfluidic device with a central vascular channel supplying the outer tumor or control chambers	ECFC-ECs, NHLFs, cancer and normal fibroblasts, MDA-MB-231, MCF-7, CRC-268, Caco-2, primary tumor organoids	Microfluidic chip with a perfused 3D microvasculature network delivers nutrients to the tumor to allow for cell proliferation, angiogenesis, tumor intravasation, and the evaluation of chemotherapeutic and anti-angiogenic drug responses	Shirure et al. (2018)
Vascularization, tumor-stroma and immune interactions	Lung cancer	Microfluidic chip with a vascular channel, LF/tumor spheroid channel and hollow channel	HUVECs, NHLFs, A459 cells, THP-1 monocytes	Tumor-on-chip model with a perfused vascular network enhancing the delivery of antitumor drugs and immune cells to the tumor spheroids	Kim et al. (2022)
Vascularization, tumor-stroma and immune interactions	Breast cancer	Microfluidic device with one cell chamber supplied by two media channels	ECs, NHLFs, MDA-MB-231 cells, PBMCs	Vascularized micro-tumors are supplied with nutrients via a complex microvasculature network that allows for the delivery of therapeutic drugs and immune cells	Hachey et al. (2024)
Hypoxia	Sarcoma	Microfluidic chip consisting of 16 independent channels with 15 wells (containing the spheroids)	SK-LMS-1 or STS117 cells	Microfluidic chip with spheroids containing a hypoxic core and demonstrating hypoxia-dependent treatment responses with the hypoxic prodrug tirapazine	Refet-Mollof et al. (2021)
Hypoxia	Breast cancer	Microfluidic device consisting of a central gel channel flanked by media and gas channels	MDA-MB-231 cells	Microfluidic chip with an established oxygen gradient across the gel channel demonstrating enhanced breast cancer cell migration under hypoxic conditions	Funamoto et al. (2012)
Hypoxia	Glioblastoma	Single chamber microfluidic device with two media side channels	U-251 MG and A-172 cell lines	Microfluidic model incorporating glioblastoma tumors with a necrotic core and treatment with NNC-55-0396 increases sensitivity of the hypoxic core; correlating with decreased levels of HIF-1*α*	Bayona et al. (2024)
pH, acidosis, hypoxia	Breast cancer	Microfluidic device with 3120 microchambers with a cell culture reservoir and a chemoattractant reservoir	SUM-159, SUM-149	Microfluidic chip demonstrating increased mesenchymal-mode migration of breast cancer cells under hypoxia and in an acidic TME, with HIF-1*α* inhibition and neutralization leading to reduced cell migration	Zhang et al. (2015)
pH, acidosis, tumor-stromal interactions	Breast cancer	Microfluidic chip with four tissue chambers and small fibrin chambers supplied by multiple media channels	MDA-MB-231 cells, skin fibroblasts	Bifurcated microfluidic device supporting two cellular microenvironments to assess the effect of pH on tumor viability and demonstrating that acid-neutralizing CaCO_3_ nanoparticles inhibit tumor cell proliferation and migration	Lam et al. (2021)
pH, acidosis, hypoxia	Bladder cancer	Multi-unit microfluidic chip supplied by perfusion channels	HUVECs, T24 cells	Microfluidic chip used to examine the energy metabolic of bladder cancer cells co-cultured with HUVECs	Zhu et al. (2016)
Tissue stiffness, desmoplasia, vascularization	Pancreatic ductal adenocarcinoma	Platform with microfluidic scaffold supplying the endothelialized scaffold lumen and the organoids	HUVECs, nHDFs, pancreatic ductal adenocarcinoma patient derived organoids	Microfluidic chip with fibroblast and cancer organoids interaction, leading to increased organoid size and collagen deposition, shows desmoplasia has an inhibitory effect on gemcitabine chemotherapy response	Benjamin et al. (2020)
Desmoplasia, tumor-stromal and immune interactions	Pancreatic ductal adenocarcinoma	Two-layer microfluidic chip with a cell and media chamber separated by a porous membrane	PSCs, U937 monocytes, MIA PaCa-2 cells or primary pancreatic cancer cells	Microfluidic chip with PDAC organoids surrounded by desmoplastic stroma and immune cells demonstrates that anti-stroma therapeutics can augment the effect of gemcitabine causing apoptosis of PDAC organoids	Haque et al. (2022)
Interstitial fluid pressure	Breast cancer, prostate cancer	3D microfluidic culture model	MDA-MB-231 or PC-3 cells	3D microfluidic culture model demonstrating how interstitial fluid pressure upregulates genes of EMT invasion in breast and prostate tumors	Piotrowski-Daspit et al. (2016)
Interstitial fluid pressure	Breast cancer	Microfluidic device containing a 3D porous collagen hydrogel	MDA-MB-231 cells	Microfluidic device enabling the characterization of changes in ECM structure in response to interstitial fluid pressure or flow	Wang et al. (2023)
Tumor-immune interactions, vascularization	Breast cancer	Microfluidic device with one cell culture channel lined by two media channels	hMVECs, MDA-MB-231 cells, primary macrophages	Microfluidic chip incorporating ECs and ECM scaffolds demonstrates that monocyte derived MMP9 promotes breast cancer cell extravasation and invasiveness	Kim et al. (2019)
Tumor-immune interactions, vascularization	Prostate cancer	Reconfigurable microfluidic system comprising separate, stackable layers	HUVECs, nHDFs, THP-1 monocytes, LNCap or C4-2 cells	Microfluidic chip incorporating prostate cancer cells shows recruitment of monocytes to the tumor with cancer-induced polarization of macrophages into pro- or anti-inflammatory	Yu et al. (2019)
Tumor-immune interactions, vascularization	Breast cancer, melanoma	Microfluidic device enclosing three rectangular compartments	HUVECs, monocytes, MDA-MB-231 or MDA-MB-435 cells	3D vascularized microfluidic model demonstrating the interaction of monocytes and tumor cells	Boussommier-Calleja et al. (2019)
Tumor-immune interactions	Liver carcinoma	Microfluidic device with central tissue channel supplied by media channels	TCR-transduced T cells, HepG2 cells	Microfluidic platform allowing for the preclinical assessment of TCR-engineered T cells against cancer hepatocytes	Pavesi et al. (2017)

Abbreviations: ECs, endothelial cells; HUVECs, primary human umbilical vein endothelial cells; EC-FCs, endothelial colony-forming cells; hMVECs, Human microvascular endothelial cells; LFs, lung fibroblasts; NHLFs, normal human lung fibroblasts; nHDFs, normal human dermal fibroblasts; NBFBs, normal breast fibroblasts; PSCs, human pancreatic stellate cells; PBMC, peripheral blood mononuclear cell; TIME, telomerase immortalized microvascular endothelial cells; EMT, epithelial-mesenchymal transition.

Besides the incorporation of tumor vasculature as a critical component of the TME, several groups have leveraged tumor chips to study the effect of metabolic stress, such as hypoxia and tissue acidosis (Zheng et al., 2021; Refet-Mollof et al., 2021). For example, Funamoto et al. developed a microfluidic platform with an established oxygen gradient across the cell channel and were able to demonstrate the enhanced migration of MDA-MB-231 cells under hypoxic conditions compared to normoxia (Funamoto et al., 2012). In a different study, Refet-Mollof et al. incorporated sarcoma spheroids with a hypoxic core into their microfluidic device to examine hypoxia-dependent treatment responses with the hypoxic-activated prodrug tirapazine (Refet-Mollof et al., 2021). Similarly, Bayona and colleagues established a microfluidic platform containing glioblastoma tumors and demonstrated that the tetralol derivative NNC-55-0396 could increase the drug sensitivity of the tumor hypoxic core, leading to increased tumor death in the platform, which correlated with decreased levels of HIF-1α *in vitro* (Bayona et al., 2024). Interestingly, Zhang and colleagues used their microfluidic platform to demonstrate that breast cancer cells show increased mesenchymal-mode migration under both hypoxic and acidic conditions of the TME, which can be reversed via HIF-1α inhibition and neutralization of the acidic microenvironment (Zhang et al., 2015). Similarly, Lam et al. assessed the effect of pH on breast tumor viability, demonstrating that acid-neutralizing CaCO_3_ nanoparticles could inhibit tumor proliferation and migration (Lam et al., 2021). In addition, microfluidic chips were used by Zhu and colleagues to examine the metabolic characteristics of bladder cancer cells co-cultured with human umbilical vein endothelial cells (HUVECs) (Zhu et al., 2016). Their study demonstrated that HUVECs rely on aerobic glycolysis, while T24 bladder tumor cells depend on oxidative phosphorylation, consistent with Sobrino et al.’s findings using fluorescence lifetime imaging microscopy (FLIM) of VMT metabolomics (Sobrino et al., 2016). Accurate metabolic coupling between both cell populations creates a positive feed-forward loop allowing for tumor growth and metastasis.

Another area of research aims to replicate physical cues within the TME, including desmoplasia, tissue stiffness and interstitial fluid pressure, in microfluidic models. For example, both Benjamen et al. and Haque et al. aimed to evaluate the effect of desmoplasia or tissue stiffness on the therapeutic response of pancreatic ductal adenocarcinoma (PDAC) tumors to gemcitabine and found that a desmoplastic stroma inhibits the effect of gemcitabine on the tumor (Benjamin et al., 2020; Haque et al., 2022). However, anti-stroma therapeutics (such as CD44 inhibitors) can augment the effect of gemcitabine; leading to significant apoptosis of PDAC organoids (Haque et al., 2022). In addition, Piotrowski-Daspit and team invented a 3D microfluidic culture model to assess how interstitial fluid pressure regulates the invasion of human breast or prostate tumors on a molecular level (Piotrowski-Daspit et al., 2016). Interestingly, interstitial fluid pressure upregulates the expression of genes linked to EMT, including vimentin and Snail, resulting in increased motility and collective invasion. Further, Wang et al. used a microfluidic device with a 3D porous hydrogel containing breast cancer tumor cells to characterize changes in ECM structure in response to interstitial fluid pressure or flow (Wang et al., 2023). Importantly, constant interstitial fluid pressure led to increased collagen remodeling by the tumor cells - characterized by enhanced collagen fibril bundling - in comparison to constant interstitial flow; highlighting the critical role that physical microenvironmental factors play in shaping tumor behavior.

Lastly, a subset of microfluidic devices enables the integration of immune cells in order to assess their complex interactions within the TME and examine new avenues for immuno-oncology-based treatment approaches. However, these approaches are still in their infancy. For instance, the microfluidic platform of Yu et al. allowed for the study of prostate cell-mediated differentiation/polarization of macrophages and distinct macrophage-mediated angiogenic processes (Yu et al., 2019). Further, Kim and colleagues’ microfluidic device incorporated a functional vasculature and further allowed for the integration of monocytes. They demonstrated that monocyte-derived MMP-9 can promote breast cancer cell extravasation and enhance cancer cell invasiveness via the destruction of endothelial tight junctions (Kim et al., 2019). In addition, Boussommier-Calleja et al. presented a microfluidic device demonstrating the interaction of monocytes and tumor cells in a 3D vascularized microfluidic model (Boussommier-Calleja et al., 2019). Notably, the researchers successfully replicated various subsets and maturation stages of monocytes (e.g., patrolling versus inflammatory monocytes) in their system. Monocytes were found to transmigrate through the microvascular networks, whereby only inflammatory CCR2+ monocytes, but not patrolling CCR2− monocytes, extravasated, which closely mimics *in vivo* behavior. Additionally, monocytes were found to directly inhibit cancer cell extravasation in a non-contact-dependent manner. However, once monocytes transmigrated through the vasculature and adopted a macrophage-like state, their effect on cancer cell extravasation was diminished. Moreover, the microfluidic platform described by Pavesi and team allows for the preclinical assessment of TCR-engineered T cells against hepatocellular carcinoma cells, that are embedded in a 3D collagen gel (Pavesi et al., 2017). Remarkably, the researchers were able to show T cell migration and killing of liver tumor cells - dependent on oxygen availability and inflammatory environment - in the device. However, this approach lacks functional microvasculature and stromal cells. Indeed, few models have successfully achieved a high level of cellular complexity and functionality, and most do not incorporate next-generation technologies.

In pursuit of advancing microfluidic platforms for tumor modeling and drug screening approaches, the integration of next-generation technologies holds immense potential (see next section). Techniques such as 3D bioprinting enable precise spatial organization of cells within microfluidic devices, facilitate the establishment of high cellular complexity and promote a more accurate recapitulation of tumor architecture and heterogeneity (Dababneh and Ozbolat, 2014; Liu et al., 2022; Fang et al., 2022; Deliorman et al., 2023). Moreover, applying single-cell analysis techniques to MPS models offers unique opportunities to dissect the cellular heterogeneity within tumors, study intra-tumoral cellular interactions and elucidate critical signaling pathways involved in tumor progression, invasiveness and therapeutic resistance (Zhou et al., 2021; Hachey et al., 2023a). Further, biosensors integrated with MPS platforms enable real-time monitoring of dynamic cellular responses and biomolecular activities within the system, advancing our understanding of cell proliferation, viability, physiology and metabolic activity under various TME conditions (Edmondson et al., 2014; Deliorman et al., 2023). In sum, integrating these next-generation technologies into tumor chips will advance our understanding of the complex processes in the TME contributing to tumor progression and will be beneficial for overcoming treatment resistance.

## 4 Next-generation technologies in MPS

In the following sections, we will highlight tumor chips that aim to integrate next-generation technologies, including bioprinting (4.1), biosensing (4.2) and next-generation sequencing (4.3), to advance cancer research and enhance our understanding of the TME (Figure 2).

### 4.1 Bioprinting

Three-dimensional cell culture models, such as spheroids and organoids, undergo non-guided spontaneous self-assembly to mimic tissue and organ development. In contrast, 3D bioprinting – the computer-guided process of printing cells, arranging components and biocompatible materials into complex, highly-organized living tissues or organs - enables precise spatial control over matrix properties, cell location and topology (Dababneh and Ozbolat, 2014; Fang et al., 2022). Hence, bioprinting improves the accuracy of the TME representation by enhancing the cellular complexity and patterning of the TME, encouraging essential cell-cell and cell-matrix interactions, and enabling designs that simulate tumor vascularization (Dababneh and Ozbolat, 2014; Fang et al., 2022; Wang et al., 2024). Despite its many advantages, challenges regarding cell viability, practicality and biocompatibility, as well as printing speed and resolution, have been encountered (Dababneh and Ozbolat, 2014). In response, extensive research has been conducted to address these challenges by enhancing bioprinters (Liu et al., 2017; Reid et al., 2019), developing innovative bioinks (Cheng et al., 2016; Highley et al., 2019; Cadamuro et al., 2023; Xu et al., 2023; Wu et al., 2024), and diversifying cell-printing techniques (Dababneh and Ozbolat, 2014; Maloney et al., 2020; Fang et al., 2022; Fritschen et al., 2024). The improvements in bioprinters focus primarily on achieving higher printing resolutions, simultaneous deposition of biomaterials, increased throughput, and affordability. Meanwhile, advancements in bioink formulations, biocompatibility, gelation predictability, and biological properties enable the creation of biologically relevant 3D tissue constructs (Dababneh and Ozbolat, 2014; Fang et al., 2022). Materials used for bioinks include alginate, gelatin, collagen, agarose, fibrin and chitosan (Gungor-Ozkerim et al., 2018). Further, many different bioprinting techniques have been developed, including the four most common cell-printing techniques: extrusion bioprinting, inkjet bioprinting, stereolithography (SLA) and laser-assisted bioprinting.

Details on the advantages, drawbacks and technical details of these techniques have been covered in other reviews (Dababneh and Ozbolat, 2014; Hagenbuchner et al., 2021; Sachdev et al., 2022). The choice of a suitable bioprinting technique is dependent on the cellular density of the tissue being created (Fang et al., 2022), the printing resolution required, and the cells’ resistance to injury or damage via shear stress or thermal and mechanical procedures (Dababneh and Ozbolat, 2014; Ning et al., 2020). Interestingly, in the last decade there has been a notable upward trend of researchers combining bioprinting with microfluidic devices and efforts to simplify the integration of both approaches have been intensified (Fang et al., 2022; Reineke et al., 2024). Importantly, this integration allows for the distribution of proteins, growth factors and cells with high spatial resolution, as well as the establishment of biological barriers, in the developing tumor tissue. Today, the combination of bioprinting and tumor chip approaches has been applied to a variety of cancers including, but not limited to, breast cancer (Hamid et al., 2014; Hamid et al., 2015; Xie et al., 2020; Lee et al., 2023), liver cancer (Hamid et al., 2015; Snyder et al., 2011; Wu et al., 2024; Liu et al., 2023), bladder cancer (Kim J. H. et al., 2021), neuroblastoma (Nothdurfter et al., 2022), glioblastoma (Zhang et al., 2016; Neufeld et al., 2021), lung cancer (Das et al., 2022; Liu et al., 2023) and CRC (Skardal et al., 2016; Vera et al., 2024). Nevertheless, microfluidic chips that integrate bioprinting technology (Table 2) remain in their early developmental stages. Many of the systems still exhibit relatively low cellular complexity and therefore do not fully leverage the potential benefits of bioprinting technology. The following section will focus on bioprinted models that demonstrate progressively higher levels of complexity by incorporating key cellular populations—such as endothelial, stromal, and immune cells—present in the tumor microenvironment (TME).

**TABLE 2:** Examples of tumor chips integrating bioprinting techniques.

Bioprinting type	Bioink	Microfluidic device	Cancer and cell type (s)	Key findings	Source
Inkjet bioprinting	Sodium alginate	Microfluidic chip with a cell chamber supplied by media channels	Liver cancer, Glioblastoma; HepG2 and U251 cells	Integration of inkjet cell printing and microfluidic chip technology to create precise cell patterns of HepG2 and U251 cells to study drug diffusion and metabolism	Zhang et al. (2016)
Extrusion bioprinting	NA	Different microfluidic chips with varying pore sizes	Breast cancer MDA-MB-231 cells	Fabrication of a three-dimensional cell-laden microfluidic chip for detecting drug metabolism via extrusion bioprinting	Hamid et al. (2014)
Extrusion bioprinting	NA	Sinusoidal microfluidic chip	Liver cancer; HepG2	Generation of an advanced microfluidic chip allowing the investigation of cancer cells in a co-cultured microfluidic environment	Hamid et al. (2015)
Inkjet bioprinting	GelMa	3D tumor array chip	Breast cancer; MDA-MB-231	Fabrication of a multi-layer microfluidic chip enabling the screening of drugs	Xie et al. (2020)
Extrusion bioprinting	GelMa	Microfluidic device with a cell chamber and microfluidic channels connected to a media chamber	Lung cancer; A549, HLF	Usage of extrusion bioprinting to recapitulate key biological and physical cues of the human lung and examine the effects of cigarette smoke extract on the metastatic potential of lung cancer cells	Das et al. (2022)
Drop-on-demand bioprinting	Agarose	Single or triple chamber microfluidic device with two fluidic channels	Liver cancer; HUVEC, HDF, HepG2	Drop-on-demand bioprinting to create a vascularized liver carcinoma on-a-chip and demonstrate liver spheroids growth via vessel-mediated support	Fritschen et al. (2024)
Alternating viscous and inertial force jetting (AVIFJ)	GelMa	Microfluidic chip with one central cell channel and two adjacent media channels	Hepatocellular carcinoma; HUVECs, HepG2 cells	Establishment of a bioprinted microfluidic chip incorporating vascularized hepatocellular carcinoma spheroids of various stages that show stage-specific drug responses	Liu et al. (2023)
Extrusion bioprinting	Porcine brain decellularized ECM and collagen	Microfluidic chip with concentric ring structure	Glioblastoma; HUVECs, U-87 cells, primary glioblastoma tumors	Bioprinting of a patient-derived glioblastoma on-a-chip model to recapitulate key pathophysiological tumor features and identify patient-specific therapies	Yi et al. (2019)
Extrusion bioprinting	Thermosensitive hydroxypropyl chitin hydrogel and Matrigel	Microfluidic chip with two channels and a single-row array of microstructures in between	Liver cancer; HUVECs, SMMC-7721 cells, PBMCs	Extrusion bioprinting to create microfluidic chip with vascularized hepatoma spheroids to assess immunotherapy response	Li et al. (2019)
*In situ* bioprinting	Skin-derived ECM bioink, alginate	Microfluidic chip with a metastatic cancer unit and a perfusable vascular endothelium system	Melanoma; SK-MEL, HUVECs, THP-1 cells	Construction of a highly controllable bladder cancer-vascular platform that allows to assess the effects of *Bacillus* calmette–guerin treatment in a microfluidic environment	Kim J. H. et al. (2021)
Aspiration-assisted bioprinting	Fibrinogen	Microfluidic chip with external pump for media flow through the endothelialized channel	Breast cancer; HUVECs, MDA-MB-231 and HDFs	Development of a dynamic-flow based 3D bioprinted multi-scale vascularized breast tumor model to assess the response to chemo- and immunotherapeutics	Dey et al. (2022)

Microfluidic devices incorporating bioprinted tissues with low cellular complexity present a robust foundation for future advancements in cancer modeling. For instance, Zhang et al. (2016) demonstrated the feasibility of integrating inkjet cell printing and microfluidic chips for spatially controlled printing of multiple cell types that better mimics the *in vivo* situation. Specifically, inkjet bioprinting in combination with a sodium alginate printing matrix was used to co-pattern hepatocellular HepG2 and glioblastoma U251 cells in the microfluidic chip in order to study drug diffusion and metabolism of the prodrug tegafur. Interestingly, the researchers showed that tegafur was metabolized by the HepG2 cells to the anti-cancer drug 5-fluorouracil, which negatively affected the growth of the U251 cells in a gradient-dependent manner. This underscores that precise micropatterning achieved through inkjet bioprinting is well-suited for drug screening applications and highlights that bioprinting can significantly reduce the labor-intensive efforts required to develop physiologically relevant microfluidic model systems. Besides this approach, several other research groups (including Hamid et al. (2014); Hamid et al. (2015); Xie et al. (2020)) have significantly contributed to the field, demonstrating the establishment and suitability of cancer cell only bioprinted microfluidic devices for drug studies.

Aiming to increase the cellular complexity and recapitulate the biological and mechanical features of the human lung, Das and colleagues used extrusion bioprinting to establish a co-culture of lung fibroblasts and A549 cancer cells inside a microfluidic chip (Das et al., 2022). The distinct advantage of using this bioprinting approach for their model is its ability to construct a multi-layered microfluidic chip that closely mimics the mechanical, biological, and physical cues of the native lung. This includes features such as an air-liquid interface for simulating inhalation and exhalation cycles, and a physical diffusion barrier composed of stromal cells. Using this model (Figure 2), the researchers successfully monitored the continuous growth of A459 spheroids, observing the formation of a hypoxic core and increased invasiveness associated with upregulated N-cadherin over extended culture periods in their device. Lastly, the effect of cigarette smoke extract (CSE) and paclitaxel were tested. CSE treatment significantly enhanced the metastatic potential via increased N-Cadherin expression in the tumor cells, increasing IL-6 expression and upregulation of α-SMA in the fibroblast population. Further, paclitaxel treatment led to decreased cell viability in a concentration-dependent manner, underlining the utility of this bioprinted model for drug screening and toxicity studies.

Another advantage of using bioprinting techniques for the establishment of 3D tumor chips is the facilitated integration of a (dys-) functional tumor vasculature, which is essential for modeling the physiological delivery of nutrients, metabolites and drugs into the tumor tissue. A recent example has been published by Fritschen and colleagues, who aimed to enhance the scalability of MPS through a combination of drop-on-demand bioprinting and robotic handling (Fritschen et al., 2024). Via the usage of multiple print heads with different bioinks, the researchers were able to create a vascularized liver carcinoma model on a microfluidic chip that incorporates organ-specific cells, connective tissue and self-assembled, complex vasculature networks. While perfusion of these vasculature networks could be demonstrated to support the growth of the HepG2 spheroids, future studies need to evaluate the suitability of this model for translational studies. In this regard, Liu and colleagues have developed a bioprinted microfluidic model of vascularized, hepatocellular carcinoma of various clinical stages and reported stage-specific drug responses and/or therapy resistance to cisplatin and bevacizumab treatment, underlining their systems potential to dissect tumor-stromal interactions for the development of effective therapeutic strategies for hepatocellular carcinoma patients (Liu et al., 2023). Another patient-oriented approach has been documented by Yi et al. (2019), who bioprinted a human glioblastoma-on-a-chip model by combining patient-derived glioblastoma tumors with vascular ECs and a decellularized extracellular brain matrix (BdECM) in their concentric-ring structure chip. This model was shown to maintain a radial oxygen gradient and recapitulate the pathological features of native glioblastoma tumors such as hypoxia-induced necrotic core formation, pseudopalisading and spatial heterogeneity. Interestingly, the BdECM bioink demonstrated a superior ability to promote angiogenesis, enhance the expression of cell junction molecules such as PECAM1 and upregulate ECM remodeling proteins like MMP9 in HUVECs, compared to a collagen matrix. Further, it more effectively promoted the invasion and characteristic spindle-like morphology of glioblastoma cells. In addition, the bioink type effected the drug sensitivity to cisplatin and a metalloproteinase-1 inhibitor of highly aggressive glioblastoma tumors. Lastly, the researchers aimed to identify patient-specific responses to concurrent chemoradiation using temozolomide (TMZ) in their chip and demonstrated differential responses that aligned with the clinical outcomes of patients. This study highlights how bioprinting technology can facilitate the construction of complex, highly biomimetic microfluidic ecosystems to evaluate the synergistic effects of the TME (e.g., BdECM, oxygen gradient, tissue structure) on glioblastoma pathophysiology.

Immune cells are an essential cellular component of the TME; however, their integration into bioprinted microfluidic models remains limited to few studies. One bioprinted microfluidic system integrating PBMCs for immunotherapy treatment has been invented by Li et al. (2019), who used extrusion bioprinting to create a tumor chip containing uniform-sized hepatoma spheroids besides a HUVEC-lined channel. In addition, a thermo-sensitive hydrogel was used during the printing process to maintain both the location and morphology of the spheroids during perfusion with media. Upon establishment of the 3D tumor system, the administration of the monoclonal antibody metuzumab (directed against CD147) led to a dose-dependent decrease in SMMC-7721 spheroid proliferation and invasion in the device. In addition, increasing concentrations of metuzumab in the presence of PBMCs led to increased cytotoxicity. Interestingly, 2D models showed a stronger ADCC effect, as well as decreased MMP-2 and MMP-9 secretion, compared to the microfluidic 3D model. This reduced drug sensitivity in 3D, as opposed to 2D, serves as an analogy to the common discrepancy between *in vitro* and *in vivo* experiments, where *in vivo* conditions typically require higher drug doses to achieve the same inhibitory effect observed in *in vitro* 2D models. A second study incorporating monocytes into their bioprinted, vascularized bladder cancer microfluidic device was performed by Kim J. H. et al. (2021). Here, bioprinting served as a means to establish a complex model to validate the immunologic effects of the TME on the tumor cells. Upon establishment of the bladder cancer-on-a-chip devices, which integrated fibroblasts, HUVECs, differentiated THP-1 cells and one of two types of bladder cancer cells (T24 and 5637), *Bacillus* Calmette–Guérin (BCG) treatment - a standard, intravesical immunotherapy for bladder cancer patients - was administered. BCG treatment led to spikes in TNFα, IL-6 and IFN-γ secretion 6 h post drug administration, indicative of the initiated immune response and correlating with directed THP-1 migration upon 24 h. Further, a dose-dependent decrease in cell proliferation and cell viability within 3 days post treatment was measurable.

Another platform employing a bioprinting approach to create a vascularized breast tumor model of high complexity for immunotherapeutic testing has been described by Dey et al. (2022). In this model, tumor spheroids were printed at varying, pre-defined distances from the perfused endothelial channel, inducing angiogenic sprouting and spheroid vascularization over the 6-day time course. Proximal spheroids exhibited a higher sprouting density compared to distal ones. Similarly, the spatial location of the tumor spheroids influenced their invasiveness, with vessel-proximal spheroids demonstrating increased invasion. In order to further validate the platform, the devices were treated with the drug doxorubicin, which induced dose dependent cytotoxicity in the tumor spheroids and stimulated the expression of VEGF, IL-6, monocyte chemoattractant protein-1 (MCP-1) and macrophage colony-stimulating factor (M-CSF). Lastly, CAR-T cells directed against HER2 and CD19 were perfused through the vascular channel, adhered within 24 h and eventually infiltrated into the tumor site. Importantly, anti-HER2 CAR-T cells were able to suppress tumor growth by up to 44%, whereby anti-CD19 CAR-T cells failed to control tumor growth within the first 24 h. Further, these cancer-immune interactions led to the secretion of cytokines and chemokines, such as IFN-γ, granzyme A, IL13, CXCL10, and MCP1; indicative of enhanced immune activation. While this study demonstrates the advantages of immunotherapy, additional research is needed to incorporate immune cells into microfluidic models and explore their interactions with other cells in the TME. This integration will help identify targetable molecular pathways and significantly improve the clinical translation of these models.

Although the models previously discussed represent only a subset of microfluidic systems utilizing bioprinting to better replicate the TME, a diverse range of other models aim to capture various aspects of the TME. These include: tumor vascularization (Cheng et al., 2019; Nothdurfter et al., 2022); integration of immune, stromal, epithelial, and lymphatic components (Snyder et al., 2011; Cai et al., 2019; Cao et al., 2019; Kim B. S. et al., 2021; Ning et al., 2022; Nothdurfter et al., 2022; Vera et al., 2024); incorporation of patient-derived cancer cells for personalized therapies and drug screenings (Mi et al., 2019; Neufeld et al., 2021; Steinberg et al., 2023); and studies of cancer metastasis to secondary tissues or bone (Skardal et al., 2016; Meng et al., 2019; Wu et al., 2024). Further, 3D bioprinting technology is used to fabricate microfluidic devices with functionalized interior structures, suitable to capture circulating tumor cells (CTCs) and enable clinical diagnosis and treatment response (Chen et al., 2020). Despite these positive developments, there remains a notable gap in bioprinted microfluidic systems that 1) aim to dissect the complex (cellular) interactions present in the TME, and 2) effectively combine tumor vascularization with novel immunotherapy and targeted therapy approaches – a combination which could offer significant benefits in cancer research and treatment efficacy.

### 4.2 Biosensors

Biosensors are versatile, now pervasive tools with applications in biomedical diagnosis, point-of-care monitoring of treatment and disease progression, environmental monitoring, forensics, drug discovery, and basic biomedical research (Bhalla et al., 2016; Ferrari et al., 2020). Examples of well-known biosensors are pregnancy tests and glucose monitoring sensors (Bhalla et al., 2016). A biosensor consists of several components including the analyte (substance of interest), the bioreceptor (molecule recognizing the analyte and generating a measurable signal), the transducer (converting the biorecognition event into a measurable signal) and a combination of electronics and displays (processing and display of results) (Bhalla et al., 2016). For the purpose of this review, the term “biosensor” will be used in a broader context, encompassing not only biosensors utilizing a bioreceptor but also chemical and physical sensors that monitor biologically relevant analytes or stimuli. However, genetically encoded (fluorescent) biosensors, which have been used to dissect cancer-mediated metabolic adaptations or evaluate the effects of high laminar shear stress on ECs (Ovechkina et al., 2021; Coon et al., 2022), will not be discussed here. Still, important key criteria for all (bio-) sensors are their selectivity, reproducibility and accuracy, robustness, linearity and sensitivity (Bhalla et al., 2016; Mou et al., 2022). In recent years, efforts to miniaturize biosensors to the micro- or nano-scale have led to significant advances enabling the detection of single molecules (Adams et al., 2008; Bhalla et al., 2016; Ferrari et al., 2020; Gonçalves et al., 2022). However, the integration of biosensing systems with microfluidic platforms, especially tumor-on-chip devices, is still in its nascent phase. Further development and optimization are needed to fully exploit their potential for studying complex biological processes and disease mechanisms in a physiologically relevant context.

The majority of models integrating biosensors with MPS focus on quantifying oxygen levels in order to evaluate the effect of hypoxia or normoxia on cancer cell behavior (Table 3). To measure the oxygen concentration in the device, two types of oxygen biosensors, namely optical and electrochemical biosensors, have been developed for MPS applications (Ferrari et al., 2020; Mou et al., 2022). While electrochemical sensors can be characterized as simple, robust and highly sensitive, they offer the disadvantage of consuming oxygen during the measurement, which may explain why optical sensors – based on fluorescence quenching – are preferred in low oxygen settings, such as when modeling tumor hypoxia (Ferrari et al., 2020). For example, Koens et al. aimed to investigate how spatiotemporal oxygen heterogeneity effects the growth and metastatic behavior of MDA-MB-231 breast cancer cells using oxygen-sensitive nanoparticles in their double-layer microfluidic device. This system could generate uniform hypoxic oxygen concentrations as low as 0.3% and could further establish a linear oxygen gradient ranging from 3% to 17% of oxygen in a short period of time (Koens et al., 2020). The oxygen-sensitive nanoparticles embedded in the gel channel served as the oxygen biosensor, whereby the intensity of phosphorescence emitted was correlated with the amount of oxygen present in the chamber. The exposure to hypoxia activated the proliferation and motility of the breast cancer cells, which was highest at uniform levels of 5% oxygen. Further, analysis of intermittent hypoxia revealed that MDA-MB-231 were able to change their migration speed in response to the surrounding oxygen environment, demonstrating increased migration speeds during hypoxic time periods. An additional microfluidic device using phosphorescence-based oxygen concentration determination and incorporating sodium sulfite as a rapid oxygen scavenger has been invented by Shirure et al. (2020). Their device consists of a central gel chamber, loaded with MDA-MB-231 cells in a fibrin gel and supplied via media channels, and two adjacent chambers for the study of hypoxia and physioxia conditions. This system, providing fine spatiotemporal control of oxygen tension, tested the impact of hypoxia on breast cancer proliferation and migration. Interestingly, again at 5% oxygen levels, MDA-MB-231 cells showed increased growth and migration, which was correlated with increased HIF-1α expression. Importantly, knockdown of HIF-1α eliminated this growth and migratory advantage. Further, HIF-1α knockdown cells grew significantly less at mild chronic hypoxia than under physioxia. Lastly, slow temporal fluctuations (>12 h) in oxygen tension differentially impacted HIF-1α-mediated proliferation and migration, underlining the distinct effect of various oxygen concentrations and patterns on breast cancer progression.

**TABLE 3:** Examples of tumor chips integrating biosensors.

Analyte	Biosensing system	Microfluidic device	Cancer and cell type (s)	Key findings	Source
Oxygen	Optical oxygen sensor; oxygen-sensitive nanoparticles (phosphorescence)	Double-layer microfluidic device with chamber flanked by media channels and two parallel gas channels above	Breast cancer, MDA-MB-231	Microfluidic device incorporating breast cancer cells showing increased migration speed in response to hypoxic conditions (maximum increase at 5% oxygen)	Koens et al. (2020)
Oxygen	Optical oxygen sensor; Oxyphor G4 dye (phosphorescence)	Three-tissue-chambered microfluidic device with media lines connected to the central chamber	Breast cancer; MDA-MB-231	Microfluidic system providing precise spatial and temporal control of O2 tension and demonstrating that temporal changes in hypoxia differentially impact HIF-1α mediated functional changes in breast cancer cells	Shirure et al. (2020)
Oxygen	Optical oxygen sensor; oxygen sensitive fluorescence dye	Multi-layer microfluidic device for culture of spheroids and mixing of five different air/nitrogen mixtures	Breast cancer; MCF7 cells; SC human monocytes/macrophages	Microfluidic device that couples breast tumor spheroids with various oxygen gradients and demonstrates increased ROS generation by MCF-7 cells due to hypoxic conditions	Fridman et al. (2021)
Oxygen	Optical oxygen sensor; NeoFox oxygen sensing system with an oxygen probe (HIOXY coating)	Microfluidic platform with a 2-layer lung cancer chamber and a liver supplied by media channels	Lung cancer (liver met); HFL-1 (fibroblasts), A549 cells, human normal liver cells	Microfluidic platform with precise control of the oxygen concentration showcasing increased HIF-1α and TGF-β1 levels in A549 cells grown under hypoxia	Zheng et al. (2021)
Oxygen, carbon dioxide/pH	Optical oxygen sensor; EOM-O2-FDM-ST-T4D-RS232-AO and PICO2 oxygen optical sensors, oxalis (oxygen alimentation system)	Variety of commercial chips: glass chamber channel 10001546 or Fluidik 1195 from chipshop or COC beflow from beonchip	Lung cancer; A549 cells	Integration of microfluidic chip with oxalis system allowing for fine control of the dissolved oxygen level and pH in the system and demonstrating hypoxia-induced gene expression changes in A549 cells	Bouquerel et al. (2022)
Oxygen, H+, glucose	Optical sensors; Image IT hypoxia green, dual-labeled pH indicator dextran, fluorescent glucose analogue	Microfluidic device with a central channel flanked by two perfusion channels	Glioblastoma; U-251 MG cells	Microfluidic chip capable of generating gradients of oxygen and demonstrating that tumor cells under hypoxic conditions switch towards increased glycolysis	Palacio-Castañeda et al. (2020)
Oxygen, glucose, ROS, apoptosis	Optical sensors; Image-iT hypoxia reagent, fluorescent glucose analogue	Microfluidic device with one cell chamber supplied by two media channels	Colon cancer, Glioblastoma; HCT-116, U-251 MG, NK cells	Microfluidic device capable of monitoring oxygen and glucose concentrations in real time and demonstrating differences in glucose uptake based on cell type and spatial location in the device	Ayuso et al. (2016)
Oxygen, lactate, and glucose	Integrated electro-chemical chemo- and biosensors, pHEMA with entrapped lactate oxidase or glucose oxidase	Microfluidic chip with two parallel cell compartments supplied by three adjacent fluid channels	Breast cancer; breast cancer stem cell line 1 (BCSC1)	Microfluidic device enabling the real-time analysis of oxygen, lactate and glucose levels and demonstrating responses to alterations in culture conditions and cancer drug exposure	Dornhof et al. (2022)
Oxygen	Optical oxygen sensor; oxygen-sensitive luminophore Ru-(Ph2phen3) Cl2 oxygen sensor	Microdevice with a base structure incorporating a cell monolayer and structures to control oxygen diffusion	Breast cancer; MCF-7 cells	Microfluidic device allowing for the establishment of oxygen gradients and demonstrating the induction of HIF-1α and Glut-1 expression	Ando et al. (2017)
Oxygen	Ratiometric oxygen sensor; oxygen sensor fibox 2, Arduino micro-controller	Gas-permeable three-layer microfluidic device with one cell channel surrounded by hydration and gas control channels	Breast cancer; MCF-7 cells	Microfluidic device with breast cancer spheroids demonstrates spheroid swelling and shrinkage in response to time-varying oxygen profiles	Grist et al. (2015); Grist et al. (2019)
Oxygen, H+	Optical oxygen and pH sensor; oxygen-sensitive fluorescent foil (SF-RPSu4), fluorescent pH indicator dye	An open-end, specialized glassware product placed on top of monolayer cells creating a narrow cell channel	Breast cancer; MDA-MB-231 cells	Microfluidic platform with cell-induced gradients of oxygen and H+ demonstrating that breast cancer cells preferentially migrate towards higher pH/oxygen regions	Takahashi et al. (2020)
H+, TEER impedance	Optical pH sensor, TEER impedance sensor (ITO-based), Arduino micro-controller system	Glass-based microfluidic chip with one cell chamber	Lung cancer; NCI-H1437 cells	Microfluidic device with integrated pH and TEER impedance sensors demonstrating that increasing concentrations of doxorubicin resulted in increased cell death compared to docetaxel	Khalid et al. (2020)
Oxygen, pH, temperature, soluble biomarkers	Label-free electrochemical immunobiosensors and physical sensors (optical pH and oxygen sensors and a temperature probe)	Microfluidic device consisting of microbioreactors, breadboard, reservoir, bubble trap and physical and electrochemical sensors	Liver cancer; HepG2/C3A, primary hepatocytes, human iPSC-derived cardio-myocytes	Heart-and-liver-cancer-chip with integrated electrochemical and physical biosensors allowing for the assessment of drug responses	Zhang et al. (2017)

Other microfluidic devices designed to study cellular responses to various levels of oxygen in the TME make use of spatially confined, oxygen scavenging chemical reactions (e.g., between pyrogallol (benzene-1,2,3-triol, C_6_H_6_O_3_) and NaOH (Shih et al., 2019). Some use the oxygen sensitive fluorescence dye tris-(2,2′-bipyridyl)-ruthenium-(II)-chloride-hexahydrate in combination with fluorescence lifetime imaging microscopy (FD-FLIM) to determine the oxygen gradient in the system. Notably, the fluorescence of ruthenium complexes is quenched in response to oxygen and follows a linear correlation trend. Ruthenium-based oxygen biosensing systems have been utilized in combination with microfluidic chips by multiple groups, including Palacio-Castañeda et al. (2020); Fridman et al. (2021). For example, Fridman’s group developed a multi-layer microfluidic device that couples the generation of MCF-7 breast cancer spheroids or co-encapsulated MCF-7-macrophage multicellular spheroids with the generation of multiple linear oxygen gradients on a single device (Fridman et al., 2021). Notably, the researchers observed hypoxia sensing of both MCF-7 and macrophages, whereby hypoxia sensing significantly increased at low oxygen levels (below 5%). Further, the oxygen concentration affected the generation of reactive oxygen species (ROS). Importantly, increased ROS production due to hypoxic conditions is associated with tumor survival and can be mediated by both tumor cells and macrophages. Interestingly, significantly increased ROS production by MCF-7 cells and macrophages was observed under 1% oxygen concentration in comparison to oxygen concentrations ranging from 5% to 20%. Finally, the researchers assessed the cytotoxicity of the anti-cancer drug doxorubicin and the hypoxia-activated prodrug tirapazamine under hypoxic conditions. MCF-7 spheroids exposed to 1% oxygen showed increased cell survival after doxorubicin treatment and decreased survival following tirapazamine treatment. These results highlight the system’s potential for future mechanistic studies and hypoxia-informed drug testing.

Optical, commercially available oxygen sensors or sensing systems to study the effect of hypoxia on lung cancer progression have been used by Zheng et al. and Bouquerel and colleagues. Specifically, Zheng et al. (2021) focused on investigating hypoxia-induced lung cancer metastasis to the liver and created a 3D-culture multiorgan microfluidic (3D-CMOM) platform incorporating HFL-1 and A549 lung cancer cells in the “lung chamber” and incorporating human normal liver cells (L02) in a separate “liver chamber” (Figure 2). After 48 h of hypoxia exposure, approximately 3.8- and 3-fold increased levels of HIF-1α could be detected in A549 and HFL-1 cells in comparison to cells cultured under normoxia, whereas L02 cells did not display any alterations in HIF1α expression. Additionally, TGF-β1 expression was increased in A549 cells grown under hypoxia, resulting in increased levels of TGF-β1 in the lung cancer chamber. Lastly, the cytotoxicity of the hypoxia-activated anti-cancer drug tirapazamine was tested. In response to treatment, the viability of A549 and HFL-1 cells was decreased in an oxygen concentration dependent manner, with 0% oxygen leading to the most prominent decrease of cell viability. The viability of L02 cells remained unchanged, underlining the ability of tirapazamine to specifically kill lung cancer cells under hypoxic conditions without harming healthy liver cells. Similarly, Bouquerel and colleagues designed a microfluidic chip incorporating A549 lung cancer cells that was connected to a novel pressure controlling system called Oxalis (OXygen ALImentation System), allowing precise control over the oxygen level in the chip (Bouquerel et al., 2022). Specifically, this system allows for the independent control of the pressure driven liquid flow rate and the concentration of dissolved oxygen, providing it with advantages over other oxygen-controlled systems. Interestingly, hypoxia-induced gene expression changes were detected in A549 cells subjected to short-term hypoxia, as they upregulated the HIF-1α target carbonic anhydrase 9 (CA9) four-fold. Lastly, this system allows the pH to be controlled via the modulation of carbon dioxide levels, enabling it to model the effects of TME acidity on tumor progression. Notably, Palacio-Castañeda and colleagues evaluated the effect of low pH on the progression of glioblastoma astrocytoma U-251 MG cells using a microfluidic chip. Using this chip, the authors introduced pH-sensitive dual-label fluorescent dextran to directly show that tumor cells switch towards increased glycolysis under hypoxic conditions, resulting in increased glucose uptake and proton accumulation.

Besides oxygen and pH, other characteristics of the TME are important determinants of cancer progression and chemotherapy resistance. Ayuso and colleagues have described a microfluidic model aimed at advancing our understanding of glucose (and oxygen) metabolism within the TME. Their device enables real-time profiling of glucose and oxygen concentrations inside the platform via a hypoxia-sensitive dye or the fluorescent glucose analogue 2-(N-(7-Nitrobenz-2-oxa-1,3-diazol-4-yl)Amino)-2-Deoxyglucose (NBDG) in combination with confocal time-lapse microscopy (Ayuso et al., 2016). The chip consists of a central channel incorporating HCT-116 or U-251 MG spheroids and is supplied by two media channels. Hypoxia was detected while monitoring the tumor chips for 4 h and could be reversed with supply of fresh media via interstitial flow. Further, when media supplemented with the fluorescent glucose analog NBDG was supplied, a defined glucose gradient in the devices containing HCT-116 cells could be detected. Interestingly, the HCT-116 cells exhausted both glucose and oxygen more rapidly than the U-251 cells, which resulted in enhanced formation of a necrotic core by HCT-116 cells. Notably, doxorubicin perfused into HCT-116 devices demonstrated complete distribution, effectively reaching hypoxic and necrotic regions, leading to significant cell death, especially close to the lateral media channels. Similar results could be obtained for U-251-MG devices treated with tirapazamine, with cell viability being lowest in the most hypoxic regions. In addition, the researchers successfully perfused an apoptosis sensing dye and a ROS-induced fluorescent compound through the device, enabling real-time quantification of apoptosis and ROS generation. Lastly, the researchers perfused activated NK cells through the device, whereby NK cells migrated from the lateral microchannels towards HCT-116 cancer cells. However, future studies will be required to evaluate the effect of hypoxia or glucose availability on NK cell behavior.

Another microfluidic platform designed to simultaneously evaluate multiple analytes, including oxygen, lactate and glucose, has been invented by Dornhof and team, who incorporated an array of electrochemical, chemo- and biosensors (Dornhof et al., 2022). Their microfluidic device allows for the formation of BCSC1 breast cancer stem cell organoids in two cell compartments supplied by three adjacent fluid microchannels. Oxygen consumption was monitored for several days and revealed that acute cyclic hypoxia did not alter oxygen consumption by BCSC1 cells based on oxygen concentration. Further, increases in glucose consumption and lactate secretion could be detected with increasing cell density. While this system is one of few models capable of evaluating multiple parameters of the TME simultaneously, future work will be necessary to address the interconnectivity of these parameters and evaluate their effect on disease progression in a more complex cellular setting. Several other tumor chips have integrated a variety of biosensors, multisensors or device-integrated biosensing materials/features, such as the studies conducted by Grist et al. (2015), Ando et al. (2017), Zhang et al. (2017), Grist et al. (2019), Khalid et al. (2020) and Takahashi et al. (2020). Nevertheless, the integration of biosensors into tumor chips is still in its early phase and predominantly limited by approaches to control or evaluate oxygen levels in the platform. In comparison, the integration of biosensors capable of measuring oxygen (Rexius-Hall et al., 2014; Shah et al., 2016), peroxide (Chmayssem et al., 2021), nitric oxide (Chmayssem et al., 2021), pH (Chmayssem et al., 2021), glucose (Bavli et al., 2016), lactate levels (Bavli et al., 2016; Chmayssem et al., 2021), and cytokines (Ortega et al., 2019), or examining tissue barrier function integrity (Shah et al., 2016; Skardal et al., 2017; Henry et al., 2017), in more rudimentary and non-cancerous microfluidic systems showcases a wider array of options.

Current efforts in biosensor-integrated tumor chips are still far from holistically modeling the TME. Most models only incorporate cancer cells and examine the effects of hypoxia on the tumor, thereby neglecting other key biochemical (cytokine or chemokine gradients) and cellular (vascularization, stromal cells and immune cells) features of the TME. Interestingly, point-of-care MPS models, designed for diagnostic purposes, represent a growing field at the intersection of microfluidics and personalized medicine. These models include biosensors for detecting the presence of circulating tumor cells (CTCs) from peripheral blood samples (Liu et al., 2013; Yan et al., 2017), identifying low expression, early cancer-specific RNA targets in human serum samples (Sheng et al., 2021), detecting circulating biomarker microRNAs (miRNAs) (Yamamura et al., 2012; Portela et al., 2020; Ishihara et al., 2021) and identifying EGFR mutations in lung tissues, performed in combination with organoid-based drug response testing (Zhang et al., 2024). While these models offer clear advantages for assisting personalized therapy decisions, they currently fail to capture the full complexity and dynamics of disease progression and treatment response over time. This underscores the need to advance tumor chips integrated with biosensors to enhance our understanding of the effect of various biological and biophysical cues in the TME.

### 4.3 Next-generation sequencing

Advancements in sequencing and instrumentation have revolutionized bioinformatic analysis, enabling the examination of large batches of cells or vesicles at high resolution. Next-generation sequencing (NGS) technologies, such as single-cell RNA sequencing (scRNA-seq) and single-cell proteomics, offer the capability to analyze gene expression and protein profiles at the individual cell level (Jia et al., 2022; Nofech-Mozes et al., 2023). High-throughput single-cell sequencing allows for the characterization of diverse cell types within tumors, including tumor cells, stromal cells, and infiltrating immune cells. This approach has deepened our understanding of microenvironmental dynamics, tumor heterogeneity, and the complex interplay between different cell types (Jia et al., 2022; Nofech-Mozes et al., 2023). Insights obtained from single-cell analysis are vital for identifying biomarkers of disease progression and treatment response, thereby guiding precise clinical decision-making for patients with malignant tumors (Jia et al., 2022). By integrating these techniques into MPS platforms, researchers can dissect the mechanisms involved in disease progression with unprecedented detail. Tumor chips serve as invaluable tools for investigating tumor evolution under controlled experimental conditions and providing a physiologically relevant platform for evaluating drug responses compared to conventional 2D cell culture models (Hachey and Hughes, 2018; Vunjak-Novakovic et al., 2021; Ingber, 2022). Given the high heterogeneity of tumors, which comprise various cell types with distinct gene expression profiles, leveraging scRNA-seq on tumor chips will enable researchers to dissect this heterogeneity, uncover rare cell populations, transitional states, and cell-to-cell variability within the tumor microenvironment (Table 4).

**TABLE 4:** Examples of tumor chips integrating cell sequencing.

Cell-cell interaction	Sequencing system	Microfluidic device	Cancer and cell type (s)	Key findings	Source
Tumor-stromal interactions	10x Genomics scRNA-seq	Microfluidic device with one cell chamber supplied by two media channels	Colorectal cancer; ECs, NHLFs, HCT116 or SW480 cells	The vascularized micro-tumor model is more like *in vivo* tumors than 2D and 3D monocultures	Hachey et al. (2021)
Tumor-stromal interactions	10x Genomics scRNA-seq	Microfluidic device with one cell chamber supplied by two media channels	Breast Cancer; ECs, normal breast tissue stromal cells, MDA-MB-231 or HCC1599 cells	Clinically relevant therapeutic targets identified at the tumor-stromal interface	Hachey et al. (2024)
Tumor-stromal interactions	10x Genomics scRNA-seq	Microfluidic device with one cell chamber supplied by two media channels	Colorectal cancer; R-VECs and patient-derived normal or tumor organoids	Interaction of R-VECs with CRC organoids led to gene clusters typical of tumor-associated vasculature	Palikuqi et al. (2020)

In pursuit of this goal, our group employed a vascularized micro-tumor (VMT) to replicate the human tumor microenvironment and accurately predict drug efficacy in a preclinical model of CRC (Hachey et al., 2021). The VMT has been shown to be a robust preclinical tool for disease modeling, drug screening, and personalized medicine applications. As briefly described in the previous section, in this system (Figure 2), ECs, fibroblasts, and cancer cells self-organize within an extracellular matrix under dynamic flow conditions, leading to the formation of a perfused vascular network that supports tumor growth and facilitates the delivery of therapeutic agents (Sobrino et al., 2016; Hachey et al., 2021; Hachey et al. 2023a; Hachey et al. 2023b). Notably, tumor growth in the VMT relies entirely on nutrient delivery through the living vascular network, closely mimicking the complex cell-cell interactions and architecture of an actual tumor. Single cell RNA-seq was performed using the 10X Genomics Chromium platform to explore gene expression variations within the dynamic 3D tumor microenvironment. The transcriptomes of cells from HCT116 CRC-containing VMTs were compared with those from matched HCT116 CRC cells, ECs, and fibroblasts cultured in 2D monolayers. Compared to cells in 2D cultures or spheroids, those in VMTs displayed a change in the distribution of tumor subpopulations, with some even being unique to the less physiologic geometries. Lineage hierarchy analysis uncovered a unique tumor population within VMTs exhibiting features of invasive CRC. Pseudotemporal reconstruction identified distinct cellular states, including the mesenchymal-like population marked by elevated expression of EMT regulatory factors (e.g., TWIST1, VIM, TIMP1, and FN1) and reduced expression of differentiation and cell adhesion markers (e.g., EPCAM, KRT8, and KRT18). Time-lapse microscopy confirmed these observations, revealing coordinated EMT events within VMTs whereby cancer cells migrated rapidly, gaining access to nearby vessels and the intralumenal space.

Through reference-based integration in Seurat, VMTs demonstrated similarity to xenograft tumors, surpassing monolayer and spheroid cultures in accurately replicating *in vivo* tumor complexity (Hachey et al., 2021). Activation of tumor-associated pathways, particularly the oncogenic Wnt pathway, was more pronounced in VMTs and xenograft tumors compared to monolayer or spheroid cultures. Moreover, gene expression and heterogeneity patterns in the VMTs resembled those of xenograft tumors, validating VMTs as suitable models for studying CRC pathogenesis. In contrast, spheroid cultures exhibited minimal gene expression alterations compared to 2D monocultures, highlighting their inability to mimic *in vivo* tumor characteristics effectively. Further validation with the SW480 CRC cell line underscored the VMT’s capacity to capture *in vivo* tumor complexity and unveil novel tumor-stromal drug targets in CRC relative to 2D or 3D cultures. Notably, VMT-derived stromal populations enriched for CAF gene signatures displayed heightened TGF-β signaling, influencing tumor growth. Targeting TGF-β signaling in stromal populations derived from VMTs with the TGF-βR1 inhibitor galunisertib specifically impeded tumor growth in the VMT model, while showing no effect in 2D or 3D monocultures. Subsequent research introduced patient-derived CRC cells into the VMT, revealing patient-specific resistance to galunisertib or the chemotherapeutic regimen FOLFOX within the VMT but not in spheroid cultures (Hachey et al., 2023b). This highlights the substantial role of stromal cells in both disease advancement and drug responsiveness, emphasizing the importance of understanding intercellular interactions within the TME to pave the way for innovative and personalized therapeutic strategies.

In a subsequent investigation, our group utilized the VMT to explore the TME in triple-negative breast cancer (TNBC) and scRNA-seq was employed to elucidate new therapeutic targets (Hachey et al., 2024). By examining cells within VMTs or vascularized micro-organs (VMOs), matched healthy tissue constructs, which incorporated primary breast tissue-derived stromal cells to mimic the tissue microenvironment, crucial signaling pathways driving tumor progression were uncovered. Additionally, it was revealed that stromal cells from normal breast tissue activate oncogenic signaling pathways within the TNBC microenvironment. Comparing interactions in VMTs with clinical data identified therapeutic targets at the tumor-stromal nexus with potential clinical relevance. Dysregulated Tie2 signaling in ECs derived from TNBC VMTs was discovered, validated clinically through Ligand-Receptor Analysis Framework (LIANA) analysis using clinical specimen datasets. Treatment of VMTs with razuprotafib, a VE-PTP inhibitor known to stabilize tumor-associated blood vessels by activating Tie2 (Shen et al., 2014; Park et al., 2016), led to enhanced tumor perfusion and increased sensitivity to low doses of paclitaxel, a standard chemotherapy drug for TNBC. Additionally, based on activated pathways identified in the scRNA-seq data, dual inhibition of HER3 and Akt demonstrated efficacy against TNBC. These findings illustrate the potential of inducing a favorable tumor microenvironment as a targeted therapeutic strategy in TNBC and showcase the utility of NGS in microfluidic models for target elucidation.

In pursuit of renewable and consistently available sources of ECs for vascularized MPS, Palikuqi et al. engineered “reset” vascular ECs, termed R-VECs, by transiently reactivating the embryonic-specific ETS variant transcription factor 2 (ETV2) in mature human ECs (Palikuqi et al., 2020). Through chromatin remodeling, ETV2 triggers tubulogenic pathways via RAP1 activation, fostering the development of vascular lumens and transforming ECs into adaptable, vasculogenic cells capable of forming functional networks both *in vitro* and *in vivo.* When introduced into a microfluidic device (termed ’Organ-on-VascularNet’), either alone or alongside malignant CRC or normal colon organoids and analyzed through scRNA-seq using the 10x Genomics Chromium platform, R-VECs co-cultured with organoids exhibited shifts in clustering patterns and gene expression compared to R-VECs cultured in isolation. While interacting with normal organoids, R-VECs demonstrated an enrichment in genes associated with EC characteristics, such as PLVAP and TFF3. Conversely, interaction with CRC organoids led to an enrichment in gene clusters typical of tumor ECs, including ID1, JUNB, and ADAMTS4, while genes associated with junctional integrity such as CLDN5 were downregulated. Notably, upon interaction with R-VECs, colon tumor cells upregulated marker genes linked to adverse prognosis and increased metastasis, such as MSLN. Collectively, these results suggest the potential of employing MPS combined with NGS to advance translational vascular medicine focused on addressing the compromised vascular niches within tumors.

In addition to advancing our understanding of cellular communication within tumors through techniques like scRNA-seq, recent research has highlighted the critical role of extracellular vesicles (EVs) in tumor biology. EVs, membrane-encapsulated structures, facilitate intercellular communication by transporting various molecules, including oncoproteins, RNA, lipids, and DNA, within the tumor microenvironment, leading to significant phenotypic changes (Xu et al., 2018). Cancer cells exhibit heightened secretion of EVs than non-cancerous cells, making them promising biomarkers for cancer diagnosis and monitoring (Dai et al., 2020). While EVs are known to be key players in cancer progression, including metastasis and premetastatic niche formation, their potential for in-depth molecular profiling remains underexplored (Dai et al., 2020). Recent studies show that EV profiling enables continuous monitoring of cancer via liquid biopsies (Domenyuk et al., 2017; Casanova-Salas et al., 2024). Microfluidic tumor chips, with their precise experimental control and small volumes, offer a unique platform for studying EVs. For instance, a recent study by Kim et al. used a 3D liver-on-a-chip model to investigate how breast cancer-derived EVs promote liver metastasis (Kim et al., 2020). The study revealed that these EVs compromise liver vascular barriers and promote cancer cell adhesion by delivering TGF1, which increases fibronectin levels. EVs from patients with liver metastasis had higher TGF1 levels and greater cancer cell adhesion, effects that were reduced by a TGF-β neutralizing antibody. This underscores the importance of EVs in metastasis and highlights the utility of MPS in studying EVs in cancer progression. Future research should focus on genomic, transcriptomic, and proteomic profiling of EV-derived molecules isolated from tumor chip models, with comparisons to patient liquid biopsies for benchmarking.

## 5 Discussion

Rapid advancements in tissue engineering have led to the development of increasingly complex and physiologically relevant microfluidic *in vitro* human tumor models. These innovations enable long-term co-cultures and hold substantial promise for deepening our understanding of cancer biology and guiding therapeutic development. However, the full potential of these models is often constrained by reductionist analytical methods. To fully realize their potential, it is essential to enhance the resolution of these assessments and integrate next-generation technologies for a more comprehensive evaluation of the TME. The future of MPS research lies in incorporating cutting-edge scientific advancements to elevate the capabilities and functionalities of these platforms. This effort requires a multidisciplinary approach, fostering collaboration across fields such as biotechnology, bioengineering, nanotechnology, and computational biology. Central to this integration is the development and refinement of MPS platforms, which serve as sophisticated *in vitro* models of human tissues and organs. These platforms are designed to replicate the physiological and biochemical properties of native human tissues, both healthy and diseased, allowing researchers to investigate complex biological processes and disease mechanisms in a controlled laboratory environment. Achieving this precision requires advanced techniques for the development and characterization of MPS platforms, while ensuring they remain user-friendly and practical for laboratory use. Advanced microfluidic technologies are crucial in enabling precise manipulation of biochemical and biophysical cues within MPS platforms, accommodating complex tissue architecture and gradients of nutrients, oxygen, and signaling molecules. This control over microenvironmental conditions allows researchers to more accurately mimic physiological tissue niches, facilitating the examination of cellular responses to dynamic stimuli and drug interventions in a controlled setting. MPS models hold significant promise for evaluating the efficacy and toxicity of new drugs and identifying patient-specific factors that may influence treatment response (Hachey and Hughes, 2018; Low et al., 2021; Liu et al., 2021). Integrating next-generation technologies successfully applied in other healthcare and research fields can enhance these systems. By combining microfluidic systems with advancements in bioprinting, biosensors, and single-cell sequencing, we can deepen our understanding of the TME and identify patient-specific factors driving disease progression and therapeutic resistance.

Apart from the technologies discussed in this review, there are numerous opportunities to further propel MPS research by integrating advanced technologies into research pipelines and/or directly into the chips themselves. The field of MPS is undergoing rapid evolution, with several exciting developments on the horizon. These advancements encompass the creation of more intricate and physiologically relevant models, the expansion of MPS applications to encompass a broader spectrum of diseases, and the integration of novel technologies into MPS models. For instance, incorporating computational modeling and machine learning algorithms enables the prediction of complex biological phenomena and drug responses within MPS platforms (Cadavid et al., 2024; Marques et al., 2024). Through the fusion of experimental data with computational simulations, researchers can gain mechanistic insights into cellular behaviors, refine experimental conditions, and identify optimal therapeutic strategies *in silico* before validation *in vitro* or *in vivo.* Advanced imaging techniques, such as multiphoton microscopy and super-resolution microscopy, provide unparalleled insights into cellular dynamics and interactions within MPS platforms, facilitating comprehensive characterization of complex biological processes (Sobrino et al., 2016). Artificial intelligence and machine learning can then be applied to the vast and complex imaging data acquired from tumor chip systems to automate image processing, data analytics, and interpretation of drug response (Urban et al., 2019). Further, integrating MPS with mechanistic modeling shows promise. Given the scale and complexity differences between preclinical and clinical settings, complementary tools in mathematical modeling, such as *in silico* models or digital twins, are crucial. These tools create virtual representations that accurately simulate the physical characteristics and functions of MPS, leveraging real-time data, simulations, and advanced computational techniques to approximate biological processes (Feng and Hedtrich, 2022). Digital twins can simulate patient-specific scenarios and, when combined with tumor chips, facilitate the testing and optimization of treatment plans (Kamel Boulos and Zhang, 2021).

While integrating computational modeling into tumor chip pipelines can facilitate the prediction of biomarkers and clinical outcomes, thereby enhancing the precision and efficacy of therapeutic interventions, personalizing patient treatment remains challenging due to the complex and partially understood factors influencing disease progression. Achieving effective diagnosis, monitoring, and treatment requires advances in biomarker discovery and the continuous optimization of therapeutic interventions to ensure precise dosing and drug selection (Shin et al., 2017; Ho et al., 2020). The integration of next-generation technologies into tumor chips can significantly advance precision medicine by improving biomarker discovery through multi-omics approaches and enabling real-time, continuous assessment of treatment responses using biosensors and imaging technologies. Specifically, as NGS technologies become more sensitive, higher in resolution, and cost-effective, their integration with tumor chips will become more feasible. Additionally, optimizing and standardizing protocols for sourcing and deriving primary cells is essential for enhancing the reliability and reproducibility of tumor chip models (Bouquerel et al., 2023). Advancements in organoid technology provide a valuable blueprint for the development of MPS systems, particularly in integrating next-generation technologies into microfluidics. This integration will address current limitations in organoid cultures, such as diffusion constraints and culture longevity, thereby improving the physiological relevance and functionality of these systems (Park et al., 2019).

To facilitate the rapid adoption and integration of next-generation technologies into MPS platforms for cancer research, several key considerations must be addressed. Research groups should focus on enhancing the utility of cancer models to enable advanced experimental analysis and improve the reliability, rigor, and reproducibility of results (Marx et al., 2016). This involves developing robust methods for standardization and quality control, potentially setting new industry benchmarks (Bouquerel et al., 2023). National and international initiatives aim to standardize MPS by establishing best practices, optimizing input cues and measurement protocols, developing flow control specifications, and standardizing chip dimensions to enhance modularity and sensor integration (Reyes and van Heeren, 2019; Silverio et al., 2022; Tomlinson et al., 2023). Integrating tumor chips into industry, academia, and clinical practice requires overcoming technical challenges to improve usability and translate preclinical research into clinical applications (Ekert et al., 2020; Jung et al., 2021; Stokar-Regenscheit et al., 2024). Open dialogue and collaboration among stakeholders are essential for this transition. Designing tumor chips for specific use cases and employing multi-omics readouts for validation can help benchmark against clinical data. Pathologists and regulatory experts can assist in defining the context of use, characterizing biological content, benchmarking tissue architecture and key markers against tissues of origin, establishing relevant endpoints, and extrapolating results to human contexts (Stokar-Regenscheit et al., 2024). Developing reproducible and consistent platforms will build confidence in new testing strategies and promote collaborations between researchers and pharmaceutical companies. The aim is to adhere to the 3Rs principles—reduce, replace, and refine animal use—and advance toward more efficient drug development methods (Ekert et al., 2020; Jung et al., 2021; Bouquerel et al., 2023; Stokar-Regenscheit et al., 2024). Notably, in 2022, the U.S. Congress passed the FDA Modernization Act 2.0, which removed the legal requirement for animal testing in drug development (Stokar-Regenscheit et al., 2024). This has been a longstanding goal in the pharmaceutical industry, and the Act’s passage has sparked further discussion on adopting new approach methodologies, including MPS, for submitting new drug applications.

For broader adoption of MPS, there is a need for greater automation and improved usability of tumor chip systems by enhancing system fabrication and design. These improvements will increase compatibility with standard analytical techniques and better address specific biological needs (Junaid et al., 2017). While tumor chip fabrication has been extensively reviewed (Ingber, 2022; Gil et al., 2023), integrating next-generation technologies requires designing hardware that accommodates these advancements. Future initiatives should focus on optimizing these integrations to enhance automation and throughput, which will expedite model validation and support their transition into precision medicine applications. Automation and scaling up of MPS systems are crucial for their adoption and commercialization, as well as translating research findings into clinical practice (Marx et al., 2016). For instance, multiplexed readouts and high-throughput screening of drug candidates within MPS platforms can assess multiple compounds simultaneously, speeding up drug discovery and reducing costs. However, current MPS applications in pharmacokinetic studies face challenges such as chip material limitations and small media volumes that hinder precise drug quantification (Abaci and Shuler, 2015). Additionally, the absence of organ-level complexity and non-physiological cell-to-volume ratios limit the direct translation of MPS findings to human applications. To address these issues, efforts are underway to integrate multiple organ-specific microfluidic devices into organ-on-chip systems (Edington et al., 2018). These multi-tissue chip technologies are designed to simulate inter-organ interactions and systemic effects, enhancing the physiological relevance and complexity of *in vitro* models and enabling more comprehensive studies of organ physiology, disease pathogenesis, and drug pharmacology (Ronaldson-Bouchard et al., 2022).

## 6 Conclusion

The integration of next-generation technologies into MPS research offers groundbreaking opportunitiesto deepen our understanding of human cancer biology, disease mechanisms, and drug responses. Advancements and wider adoption of these technologies are expected to advance various aspects of oncology, such as discovering new therapeutic targets, elucidating mechanisms of resistance, and identifying diagnostic or predictive biomarkers. This progress will significantly enhance the field of precision oncology. Additionally, integrating these technologies will accelerate the validation of tumor chips, promoting their broader adoption into industry. By leveraging these advanced tools, researchers can create more accurate, predictive, and clinically relevant *in vitro* models, thereby accelerating biomedical research and translating these efforts into precision healthcare.
